# Cutin:cutin-acid endo-transacylase (CCT), a cuticle-remodelling enzyme activity in the plant epidermis

**DOI:** 10.1042/BCJ20200835

**Published:** 2021-02-24

**Authors:** Anzhou Xin, Yue Fei, Attila Molnar, Stephen C. Fry

**Affiliations:** 1 The Edinburgh Cell Wall Group, Institute of Molecular Plant Sciences, The University of Edinburgh, Edinburgh EH9 3BF, U.K.; 2 Institute of Molecular Plant Sciences, The University of Edinburgh, Edinburgh EH9 3BF, U.K.

**Keywords:** cell expansion, cuticle, cutin re-modelling, pea epicotyl, plant epidermis, transacylase activity

## Abstract

Cutin is a polyester matrix mainly composed of hydroxy-fatty acids that occurs in the cuticles of shoots and root-caps. The cuticle, of which cutin is a major component, protects the plant from biotic and abiotic stresses, and cutin has been postulated to constrain organ expansion. We propose that, to allow cutin restructuring, ester bonds in this net-like polymer can be transiently cleaved and then re-formed (transacylation). Here, using pea epicotyl epidermis as the main model, we first detected a cutin:cutin-fatty acid endo-transacylase (CCT) activity. *In-situ* assays used endogenous cutin as the donor substrate for endogenous enzymes; the exogenous acceptor substrate was a radiolabelled monomeric cutin-acid, 16-hydroxy-[^3^H]hexadecanoic acid (HHA). High-molecular-weight cutin became ester-bonded to intact [^3^H]HHA molecules, which thereby became unextractable except by ester-hydrolysing alkalis. *In-situ* CCT activity correlated with growth rate in *Hylotelephium* leaves and tomato fruits, suggesting a role in loosening the outer epidermal wall during organ growth. The only well-defined cutin transacylase in the apoplast, CUS1 (a tomato cutin synthase), when produced in transgenic tobacco, lacked CCT activity. This finding provides a reference for future CCT protein identification, which can adopt our sensitive enzyme assay to screen other CUS1-related enzymes.

## Introduction

Cutin is an extracellular polyester in the shoot epidermis of land-plants, located between the primary cell wall and the surface wax [1,2], and recently also detected in root-caps [3]. It typically constitutes 40–60% of the dry weight of the cuticle, varying between species [4,5,6]. It is widely believed to have been acquired by streptophytic plants during their migration from freshwater (charophytic green algae) to land (bryophytes) [7], which probably occurred in the Cambrian [8], and indeed there is no evidence for cutin in modern charophytes [9,10]. Cutin occurs throughout the Embryophyta, from non-vascular (e.g. the moss *Physcomitrella patens*) [11] to vascular plants (e.g. *Arabidopsis thaliana* and *Solanum lycopersicum*) [12,13], although the composition varies between taxa and even between organs in the same plant [14].

Chemically, the core structure of cutin is an aliphatic polyester of hydroxy-fatty acids (HFAs) [15,16]. The degree of polymerisation of cutin remains elusive probably owing to the difficulty of dissolving it intact. In most aerial organs, e.g. in the leaf and fruit of tomato, the major HFA is 10,16-dihydroxyhexadecanoic acid (diHHA) [14]. 16-Hydroxyhexadecanoic acid (HHA) is usually also present [17]. Where diHHA predominates, branched cutin structures can form in which both the hydroxy groups are involved in ester bonding [16,18,19,20]. In addition, epoxy-containing HFAs, fatty alcohols and aldehydes, glycerol and phenolics (e.g. *p*-coumaric acid and ferulic acid) are building blocks of cutin [14,17,21]. In a few plants, including arabidopsis, a major component of the epidermal polyester is octadecadien-1,18-dioic acid [12,22], although such components are more usually a feature of suberin rather than cutin [23].

Cutin's precursors are 2-monoacylglycerols (2-MAGs), which are biosynthesised in the endoplasmic reticulum [24] and polymerised into cutin in the apoplast [13]. Besides hydrolysis by cutinase [25], cutin polymerisation is the only known apoplastic cutin reaction, mediated in tomato fruit by cutin synthase (CUS1, previously named CD1; immunolocalised to the cuticle) [13,26]. CUS1 is a ‘GDSL’ enzyme [13], i.e. an ‘esterase/lipase/transacylases’, named from a conserved Gly–Asp–Ser–Leu (or similar) sequence near the *N*-terminus. In the polymerisation reaction, 2-MAG molecules act as acyl donors, the HFA moiety being transferred to a hydroxy group of a growing cutin molecule (to elongate cutin) or to another 2-MAG molecule (forming a dimer to initiate a cutin molecule) [13]. Notably, an HFA molecule can only be the donor in this way if the HFA's carboxy group has first been activated in some way — in this case by ester-bonding to glycerol.

Physiological roles of cutin plus its associated waxes are to minimise desiccation in the terrestrial environment [27,28,29], to protect against ultraviolet radiation (thanks to minor phenolic constituents) [30], and to provide mechanical protection against attempted microbial penetration [27,28,31,32]. Cutin also offers some protection against hydrophilic herbicides [33,34].

Besides defence against external stresses, cutin, as a part of the cuticle, also prevents inappropriate organ fusion during normal plant development [35,36]. It can also constrain seed germination by restricting imbibition [37] and it can act as a physical barrier against penetration of the stigma by pollen tubes [38].

In addition to the above roles, there is evidence suggesting that cutin may contribute as a ‘skin’, limiting the expansion of aerial organs. The epidermis certainly plays a key role in limiting the expansion of aerial organs such as stems and leaves [39,40]. In rice coleoptiles, cutinase-treatment promoted elongation [41], suggesting a specific role for cutin as a mechanical constraint to organ expansion. In agreement with this idea, removal of tomato fruit epidermal polysaccharides (but not cutin) with anhydrous hydrogen fluoride showed that cutin determines the viscoelastic behaviour of the ‘cuticular membrane’ [42]. In [43], the authors also postulated that the cuticle has sufficient tensile strength to play a role in limiting the extensibility of the epidermis. Furthermore, in sweet cherry fruit, the period 40–85 days after full bloom is associated with no net change in cutin content (∼1 mg/fruit), but a trebling in fruit surface area [44], suggesting that an existing cutin network can undergo molecular rearrangements to accommodate expansion without tearing. A genetic cutin deficiency (*cus1*, i.e. *cd1*) in tomato fruit increases the area and radial width of the epidermal cells [45], as expected if cutin limits cell expansion in certain dimensions. The same mutant had previously been reported to affect the cuticle's biomechanical properties such as Young's modulus (as expected if cutin has a structural role), although not promoting total fruit expansion [28]. The lack of effect of cutin deficiency on whole-organ growth, however, does not preclude cutin from playing a growth-limiting role; on the contrary, it is possible that, in the wild-type, cutin loosening is necessary (but usually sufficient) for normal fruit expansion, and unnecessary in the cutin-deficient mutant. This hypothesis can only be tested when the proposed cutin-restructuring enzyme has been detected and identified, such that it can be investigated genetically.

We therefore explored the hypothesis that a cutin-loosening mechanism is present during rapid growth; such a mechanism may have previously been overlooked owing to inadequate analytical techniques. In the present work, we developed highly sensitive radiochemical assays by which to investigate cutin restructuring reactions. Specifically, we pioneered a method to test whether transacylases exist that can ‘cut and paste’ cutin molecules, comparable to polysaccharide cutting and pasting by a transglycanase, xyloglucan endotransglucosylase (XET) [46], which acts during the assembly and later restructuring of the plant cell wall [47,48]. In principle, such transacylases could transiently loosen the cuticle, permitting organ growth, and afterwards restore the strength of the expanded cuticle — as may happen in sweet cherry fruit (see above).

**Figure 1.**
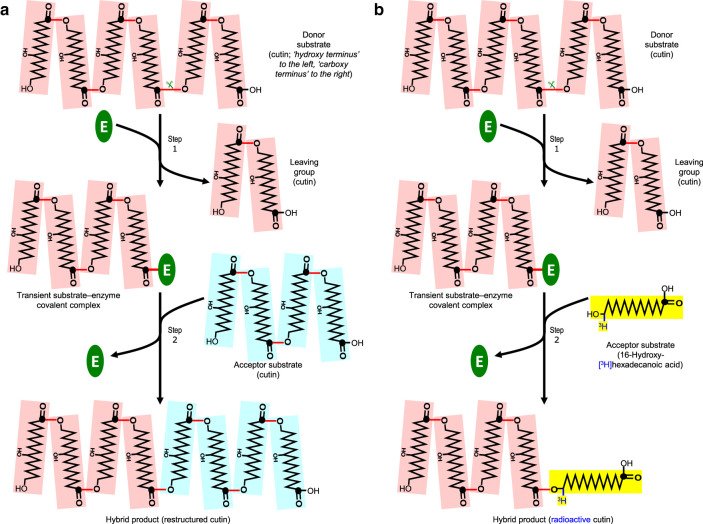
The proposed cutin:cutin transacylation mechanism. (**a**) The reaction as proposed to occur naturally *in vivo*. In step 1, a cutin chain (donor substrate, pink) is cut (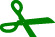) by a transacylase (**E**), forming a transient cutin–enzyme covalent complex and releasing the carboxy-terminal portion of the cutin as a leaving group. In step 2, after incremental rearrangement of the cuticular network, the enzyme-bonded portion of cutin is transferred onto a free –OH group of a neighbouring cutin molecule (acceptor substrate, pale blue), restoring the strength of the cutin and releasing the enzyme for the next cycle. (**b**) As (a) but showing the reaction occurring *in situ* when an exogenous soluble radiolabelled acceptor substrate (16-hydroxy-[^3^H]hexadecanoic acid, shown in yellow) is infiltrated into epidermal walls containing endogenous enzyme plus endogenous insoluble cutin. An insoluble radiolabelled ‘hybrid’ product is formed, which is assayed by scintillation counting. Key: ⬤, carbon-1 of a hydroxy-fatty acid moiety; red line (**—**), ester bond; (**E**), transacylase. For simplicity, the cutin molecules depicted are small, unbranched and composed entirely of 10,16-dihydroxyhexadecanoic acid residues; however, natural cutin is likely to be branched via the 10-hydroxy groups and to be of higher degree of polymerisation than shown. The cutin chains are drawn with the ‘hydroxy-terminus’ to the left and the ‘carboxy-terminus’ to the right.

CUS1, the only known apoplastic cutin-acting transacylase, requires an HFA-glycerol ester (2-MAG) as its acyl donor substrate. Our assays, in contrast, sought to detect a different type of reaction in which the donor is polymeric cutin (Figure 1a). To our knowledge, this reaction has not previously been tested for.

To describe this putative reaction, we propose the terms carboxy- and hydroxy-terminus for cutin structures. In an idealised model of cutin composed primarily of diHHA residues (Figure 1), there is a single carboxy-terminus, many mid-chain residues, and one or more hydroxy termini (in unbranched or branched cutins, respectively). The transacylation reaction envisaged (Figure 1a) could be catalysed by a cutin:cutin transacylase (CCT) — an activity not yet assayed in any protein. Such an enzyme would cleave an existing ester bond in a cutin molecule, the newly generated cutin carboxy-terminus transiently forming an ester bond to the enzyme (Figure 1a). After incremental repositioning of the cutin polymers within the cuticle, the cutin–enzyme complex would then serve as an acyl donor, transferring a segment of the cutin molecule onto a free –OH group on a neighbouring cutin chain, to create a new ester bond. The other part of the cleaved cutin molecule, now possessing a new hydroxy-terminus, would be released as the leaving group. Figure 1a illustrates only unbranched and relatively small cutin molecules, though branched or larger ones could equally participate in CCT reactions.

In the light of this background, we are exploring the breadth of cutin-related reactions catalysed by plant enzymes, helping to understand — and potentially provide exciting future ways to adjust — the plant's interface with its atmospheric environment as well as the mechanical limit to the expansion of stems, leaves, flowers and fruits.

## Materials and methods

### Chemicals and enzymes

The chemicals used for this research were mainly obtained from Sigma–Aldrich (Gillingham, U.K.; https://www.sigmaaldrich.com/united-kingdom.html), Fisher Scientific (Loughborough, U.K.; https://www.fishersci.co.U.K./gb/en/home.html), VWR (Lutterworth, U.K.; https://uk.vwr.com/store/), Thermo Scientific (Gloucester, U.K.; https://www.thermofisher.com/hk/en/home.html) and Merck (Feltham, London, U.K.; https://www.merckgroup.com/uk-en). [^14^C]Hexadecanoic acid (HA) was purchased from ARC (U.K.) Ltd. (Royston, Herts, U.K.; http://www.arcincusa.com). [1-^3^H]GalA_8_-ol (reductively tritiated octasaccharide of homogalacturonan) was from EDIPOS (Edinburgh, U.K.; http://fry.bio.ed.ac.uk//edipos.html). [16-^3^H]HHA and Me_8_-[1-^3^H]GalA_8_-ol (fully methylesterified [1-^3^H]GalA_8_-ol) were prepared as described in Supplementary Figures S4, S5, respectively. The prepared [^3^H]HHA co-chromatographed with authentic stained non-radioactive HHA, confirming its identity (Supplementary Figure S3; *R*_F_ 0.34–0.35 on TLC).

### Plant epidermis sources

*Hylotelephium spectabile* (ice plant) from an Edinburgh garden was greenhouse grown in a potting mixture [150 l medium-grade peat + 40 l horticultural sand + 560 g garden lime + 225 g ‘Osmocote Exact 5–6 Months’ slow-release fertiliser tablets + 60 g Bayer ‘Exemptor’ (insecticide containing 10% w/w thiacloprid)] with 16 h light (21°C) and 8 h dark (18°C). Seeds of *Pisum sativum* (pea; cv. meteor) and *Solanum lycopersicum* (tomato, cv. Alisa Craig) were obtained commercially. Wildtype and *cus1*-knockout tomato seeds (cv. M82) were kindly donated by Prof. J.K.C. Rose (Cornell University, U.S.A.). Pea seedlings were grown in thoroughly watered vermiculite in continuous darkness at 25°C and epicotyls harvested at 4–10 days. Tomato plants were greenhouse grown in the above potting mixture.

Epidermis containing endogenous enzymes and cutin was isolated from plants by manual peeling (*Hylotelephium* leaves and tomato fruit) or by rolling under a glass rod to extrude internal (non-epidermal) tissues and epidermal protoplasm (pea epicotyl), then immediately frozen at −80°C, rupturing the plasma membranes. Epidermis was thawed on ice before use, then washed in cold reaction buffer [25 mM succinate (Na^+^), pH 5.5; 35 ml per (maximum) 5 g fresh weight; 1 h at 4°C], which will remove intracellular metabolites including ATP, CoA and free glycerol, yielding ‘native epidermis’. During the 1 h washing, any ATP and CoA in the frozen/thawed plant material would be highly susceptible to hydrolysis by phosphatases and phosphodiesterases. Furthermore, the washed epidermis was blot-dried before assays, further removing any traces of coenzymes. Alternatively, for denatured controls, the epidermis was incubated in the water at 100°C for 1 h.

### *In-situ* CCT enzyme assays

Native epidermis was briefly blotted dry and cut into small pieces, then incubated with 0.25–12 kBq of radiolabelled substrate (e.g. [^3^H]HHA) in the 25 mM pH 5.5 buffer (routinely 6 ml per g fresh weight) at 20°C for up to 24 h. All enzymic reactions were stopped by addition of 10 volumes of freshly prepared methanol/formic acid/water (MFW) (9 : 1 : 1, v/v/v). To remove unincorporated alcohol-soluble radioactivity, we dried the epidermis onto Whatman No. 1 paper, which was run as a ‘chromatogram’ in MFW for 5–9 days.

### *Ex-situ* CCT assays with native plant enzymes

Denatured epidermis (containing cutin but no active endogenous enzymes) was washed in acetone for 3 × 1 h followed by boiling chloroform for 90 s (which removes wax)[49], then chloroform/methanol (CM) (2 : 1, v/v) at 20°C for 16 h. All samples were finally re-dried from acetone.

An enzyme mixture dominated by apoplastic enzymes was extracted from freshly rolled (=freed of protoplasm) pea epidermis by grinding in liquid nitrogen then homogenising in extraction buffer [350 mM succinate (Na^+^), pH 5.5, with 1% v/v Triton X-100 and 1% w/v polyvinylpolypyrrolidone] at 2 ml/g fresh weight with a pinch of acid-washed sand (Bio-Rad) and shaking at 4°C for 2 h. The suspension was then centrifuged at 4°C for 15 min at 3000×***g***. The supernatant (100–300 µl), containing solubilised enzymes, was incubated with the denatured epidermis (2–10 mg thoroughly dried epidermal particles) together with 0.25–1 kBq [^3^H]HHA or [^14^C]HA at 20°C for up to 24 h. Reaction termination and wash procedures were as for *in-situ* experiments. The following metal salts were added to the enzyme extract in some cutin-to-[^3^H]HHA transacylation experiments: CaCl_2_, MgCl_2_, MnCl_2_, FeSO_4_, NiSO_4_, ZnCl_2_, CoCl_2_ and KCl (final concentration 10 mM).

### *Ex-situ* CCT assays with heterologously produced plant enzyme (CUS1)

Particles of denatured epidermis (10 mg; thoroughly dried; prepared as above) containing cutin but no active endogenous enzymes were incubated with 1 kBq [^3^H]HHA in 300 µl buffer with or without 1 µg of purified His_6_-tagged tomato CUS1 protein [heterologously produced in *Nicotiana benthamiana* and purified on a cobalt column; Supplementary Figure S6] at 20°C for 1 day. Reaction termination and washing procedures were as above.

### Quantifying radioactive CCT product

For measurement of total incorporation, the washed and re-dried epidermis was added to 2 ml of homemade scintillation fluid (0.5% w/v PPO and 0.05% w/v POPOP in toluene; chosen to avoid the organic bases used in many commercial scintillants, which may cleave esters), mixed overnight, and assayed for ^3^H in a Beckman scintillation counter. For solutions or wet samples, the specimen was mixed with 10 volumes of OptiPhase HiSafe (Fisher) scintillation fluid before scintillation counting.

### Qualitative analysis of radioactive CCT product

**Figure 2.**
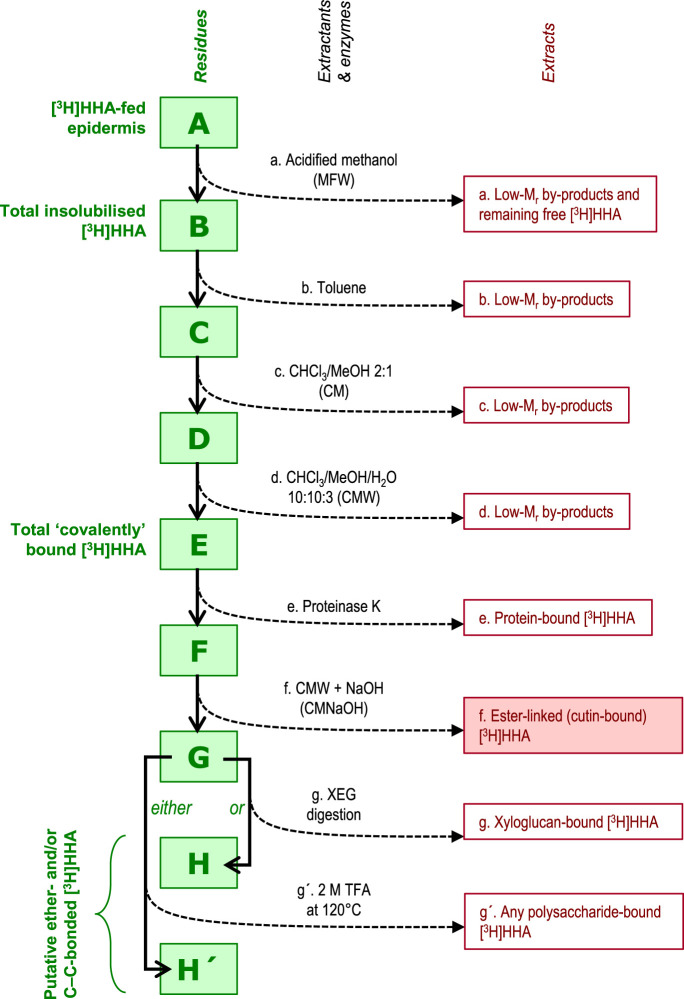
Workflow of *in-situ* CCT product characterisation. Frozen/thawed epidermis was incubated with [^3^H]HHA for up to 24 h, then the insoluble material (**A**) was treated with a sequence of extractants and enzymes, yielding a series of insoluble residues (**B**–**H**), some of which (**B** and **H**) were assayed for radioactivity. The material solubilised at each step (extracts a–g′) was also assayed for ^3^H.

To look more specifically for transacylase products, we devised a sequential extraction/degradation strategy (Figure 2). The [^3^H]HHA-fed epidermis was MFW-washed ‘chromatographically’ as above, then the insoluble material (B in Figure 2) was suspended successively in 1.0–1.5 ml toluene, chloroform/methanol (CM, 2 : 1, v/v), and chloroform/methanol/water (CMW, 10 : 10 : 3, v/v/v) [each at 20°C for 1 d], then 1 ml of 3 U/ml proteinase K from porcine liver (Sigma) in pH 8.8 buffer [50 mM ammonium (acetate^-^) containing 0.5% chlorobutanol] at 37°C for 3 days. Supernatants were collected for later analysis. To look for cutin-to-[^3^H]HHA transacylase products in the remaining insoluble material, we cleaved ester bonds in residue ‘F’ with 1.0–1.5 ml of chloroform/methanol/4 M aqueous NaOH (CMNaOH, 10 : 10 : 3, v/v/v) at 20°C for 1 day, collected the clear solution and acidified it with acetic acid (5 mol per mol NaOH). Phase partitioning to remove hydrophilic solutes was performed by a modified lipid extraction procedure [50]: 0.8 volumes of aqueous 154 mM NaCl was added gently to the CMNaOH/acetic acid solution followed by several inversions, and the upper aqueous phase was removed after centrifugation (720×***g***); then a further 0.8 volumes of 77 mM NaCl in 50% v/v methanol was added and the upper aqueous phase was removed as before. The remaining lower, chloroform-rich layer was dried and re-dissolved in MFW. Finally, to look for possible glycosidically bonded radioactivity, we digested the alkali-resistant residue (G) in 1 ml of either 0.1% (w/v) XEG [in pyridine/acetic acid/water, 1 : 1 : 98, v/v/v, pH 4.5] at 20°C overnight, or 1.0–1.5 ml of 2 M TFA (120°C for 1 h).

An aliquot of each extract mentioned above was assayed for ^3^H, and the rest was analysed by thin-layer chromatography (TLC) on plastic-backed silica gel 60 plates (Merck) in toluene/acetic acid, 9 : 1 v/v, with three ascents. The ^3^H profile was recorded on an AR2000 radioisotope scanner (LabLogic, Sheffield, U.K.) or by scintillation counting of pieces cut from the TLC plate.

### CCT activity calculation and statistical analysis

CCT products are expressed as Bq of alkali (CMNaOH)-releasable radioactivity per kBq of supplied [^3^H]HHA per 50 mg fresh weight of epidermis. Statistical analysis was by Student's *t*-test. The counting efficiency of ^3^H in epidermal samples was ∼7%.

### Methods used for supplementary data

#### Chromatographic analysis of by-products generated from [^3^H]HHA during methanolic extraction

(Supplementary Figure S3) [^3^H]HHA (dissolved in ethyl acetate) was dried in two portions. One was re-dissolved in 10 µl of DMSO and adjusted to 10 kBq/ml by addition of 25 mM succinate (Na^+^), pH 5.5; the other was redissolved at 10 kBq/ml in identical buffer (without DMSO).

To mimic the cutin-to-[^3^H]HHA *in-situ* assays, we incubated the solutions at 20°C for 1 day. The sample lacking DMSO received 1 ml of MFW (9 : 1 : 1, v/v/v). The sample that contained DMSO was further divided into four aliquots, to which we added 1 ml of a freshly prepared ‘MFW’ mixture (‘MFW’ ratios, by vol., 9 : 1 : 1, 9 : 0 : 2, 0 : 1 : 10 and 0 : 0 : 11). All five solutions were incubated for further 16 h at 20°C. They were then dried in SpeedVac, and re-dissolved in ethyl acetate and analysed by TLC on plastic-backed silica-gel plates in toluene/acetic acid (9 : 1) with three ascents of the solvent. The ^3^H profiles were recorded on an AR2000 radioisotope scanner (LabLogic, Sheffield, U.K.).

The by-product peak (*R*_F _= 0.65–0.69, co-migrating with the position of 16-oxo[16-^3^H]hexadecanoic acid (OHA) as estimated from the *R*_F_ of the intermediate used in [^3^H]HHA preparation) was detected in all the samples, indicating that none of the solvents specifically generated it. However, [^3^H]HHA from the same stock but not dried in the SpeedVac did not show the by-product. We therefore conclude that OHA is generated from HHA during drying in the SpeedVac.

#### Chemical synthesis of [^3^H]HHA

(Supplementary Figure S4) The synthesis was achieved via a two-step procedure —

*step 1:* The primary alcohol group of non-radioactive HHA was oxidised with pyridinium chlorochromate (PCC) to form the aldehyde, OHA:

$$R\text{-}\text{C}{\text{H}}_{2}\text{OH}+\text{PCC}\to R\text{-}\text{CHO}$$

*step 2:* The aldehyde group was then reduced back to the alcohol with tritiated sodium borohydride:

$$R\text{-}\text{CHO}+\text{Na}{\text{B}}^{3}{\text{H}}_{4}\to R\text{-}\text{C}{\text{H}}^{3}\text{HOH}$$

The PCC:HHA ratio for step 1 was optimised in a preliminary run [Supplementary Figure S4a; analysis by TLC on an aluminium-backed silica-gel plate in toluene / acetic acid (9 : 1), with one 105 min ascent; spots were stained by spraying lightly with 20% (v/v) H_2_SO_4_ in methanol, and heating in an oven at 100°C]. Then, under optimised conditions, the procedure was scaled up. Commercial HHA (10 mg) plus PCC (32 mg) were incubated in 1 ml chloroform at 20°C for 90 min, then at 30°C for 30 min. Any remaining PCC was then destroyed by addition of 0.5 ml methanol and the products were subjected to preparative TLC on silica-gel in toluene/acetic acid (9 : 1). *R*_F_ values were HHA, 0.31; OHA, 0.42. After a guide strip had been stained with I_2_, the bulk of the OHA was eluted in 2 ml of ethyl acetate, dried in a glass vial and resuspended in 10 µl of 10 M ammonia; 100 µl of a solution of NaB^3^H_4_ (100 MBq; 38 MBq/µmol) was added and after incubation at 20°C for 72 h, 600 µl methanol/acetone (2 : 1) was added. To remove NH_3_ and the solvents, we left the vial open in a fume hood overnight. The dry residue was then redissolved in 200 µl propan-2-ol and run by TLC under the same conditions. The [^3^H]HHA was located by autoradiography (Supplementary Figure S4b), then the relevant zone (blue rectangle) was eluted in ethyl acetate (yield 3.38 MBq, specific activity ≈ 19 MBq/mmol).

#### Chemical synthesis of methylesterified [^3^H]GalA_8_-ol

(Supplementary Figure S5) Dried [^3^H]GalA_8_-ol (4 kBq; from EDIPOS, Edinburgh, U.K.; http://fry.bio.ed.ac.uk//edipos.html) was re-dissolved in 100 µl 50 mM MES (Na^+^), pH 6.5, and 100 µl methanol was added. NHS/EDC reagent comprised 45 mg dry EDC dissolved in 1 ml of freshly prepared 0.3% w/v NHS in methanol/MES (1/1); this whole 1 ml of solution was then immediately added to the [^3^H]GalA_8_-ol solution. After 16 h incubation at 20°C, 100 µl acetic acid was added and the reaction mixture was electrophoresed (alongside pure [^3^H]GalA_8_-ol as external marker) on Whatman no. 3 paper in pH 6.5 buffer (acetate/pyridine/water, 1 : 33 : 300) at 3 kV for 90 min [51]. After scanning on an AR2000 radioisotope scanner (LabLogic, Sheffield, U.K.), the neutral fraction (Me_8_-[^3^H]GalA_8_-ol) was eluted from the paper in 50% methanol, dried, redissolved in water and stored frozen.

#### Heterologous expression of SlCUS1 in *N. benthamiana*

##### Extraction and purification of RNA

(Supplementary Figure S6) Extraction of nucleic acid was according to [52], Pericarp from 2 cm tomato fruits (M82; 15 days after anthesis) was homogenised to a fine powder by punching and grinding in liquid nitrogen. The ground pericarp (150 mg) was vortexed vigorously with 600 µl pre-cooled extraction buffer [0.1 M glycine (Na^+^), 10 mM EDTA, 0.1 M NaCl, pH 9.5] containing 2% (w/v) SDS, followed by addition of 600 µl phenol solution [∼90%, saturated with 10 mM aqueous Tris (Cl^–^), pH 8.0]. The mixture was centrifuged at 20 000 *g* at 4°C for 10 min, then the upper layer was mixed with 600 µl phenol solution/CHCl_3_/3-methylbutan-1-ol (25 : 24 : 1, v/v/v) and centrifuged as before. This step was repeated. The upper layer was mixed with an equal amount of CHCl_3_/3-methylbutan-1-ol (24 : 1, v/v) and centrifuged as above. The upper layer was mixed with 2.5 volumes of absolute ethanol plus 0.1 volumes of 3 M acetate (Na^+^, pH 5.2) and left at 0°C for 30 min, precipitating total nucleic acids. After centrifugation as before, the supernatant was discarded, and the pellet was washed in 80% ethanol then dried in a stream of air and redissolved in DEPC-treated water (Thermo Fisher Scientific).

TURBO DNA-*free*^TM^ kit (Thermo Fisher Scientific) was used for removal of DNA. RNA was re-precipitated by addition of 2.5 volume of 80% ethanol plus 0.1 volumes of commercial “3 M sodium acetate” (adjusted to pH 5.5 with acetic acid); the mixture was stored at 0°C for 30 min, centrifuged at 4°C for 5 min and the supernatant discarded. Remaining salts were removed by washing in 80% ethanol, followed by centrifugation at 4°C for 5 min. The pure RNA was redissolved in 15 µl DEPC-treated water.

##### mRNA Reverse transcription and cDNA amplification

*CUS1* cDNA was synthesised with the “SuperScript® III First-Strand Synthesis kit” (Thermo Fisher Scientific) using a gene-specific reverse primer S1_CUS1_noStop_rev. We then amplified the corresponding cDNA harbouring the AgeI (5′) and SmaI (3′) cutting sites (underlined), without the stop codon, using the primers S1_CUS1_ATG_for (TTTACCGGTATGGCCACACCTACTATTATTTTGAG) and S1_CUS1_noStop_rev (AATCCCGGGTGCATGTGAATCCATAGCCAG) using Q5® High-Fidelity DNA Polymerase (NEB)’. Amplification conditions were: initial denaturation (30 s at 98°C), followed by 35 cycles of denaturation (5–10 s at 98°C), annealing (30 s at 66°C), extension (30 s at 72°C), and finally extension (5 min at 72°C). The CUS1 cDNA was purified from the 1% agarose gel with the “QIAquick Gel Extraction kit” (QIAGEN).

##### CUS1 heterologous expression in *N. benthamiana*

The vector, pEAQ-*HT* [53], used by [13] to heterologously express C-terminal His_6_-tagged CUS1 was kindly donated by Prof. George Lomonossoff (John Innes Centre, U.K.).

*E. coli* (DH5α) was chemically transformed at 42°C for 1 min with pEAQ-*HT*. After growth on LB agar with 50 µg/ml kanamycin at 37°C overnight, pEAQ-*HT* was then harvested with a “QIAprepSpin Miniprep kit” (QIAGEN). The SmaI and AgeI double-digested [13] pEAQ-*HT* was ligated with the identically digested CUS1 cDNA following the “T4 DNA Ligase” protocol (NEB). The pEAQ-*HT*::CUS1 construct was introduced into *E. coli* by transformation and extracted as before.

Transformation of *Agrobacterium tumefaciens* with the construct and infiltration into *N. benthamiana* was as described by [53], except that YEP medium was used for *A. tumefaciens* strain GV3101 and *N. benthamiana* leaves were harvested 5 days after infiltration.

##### His_6_-tagged CUS1 purification by immobilised cobalt affinity chromatography

*Agrobacterium*-infiltrated *N. benthamiana* leaves were harvested, ground in liquid nitrogen and stored at −80°C. Protein extraction was as described by [13], except that leaf weight and the pre-cooled extraction buffer were mixed at a 1 : 3 (w/v) ratio at 4°C and shaken vigorously at 4°C for 30 min with the addition of 0.5 mM phenylmethylsulphonyl fluoride and 1 mM 2-mercaptoethanol. The final supernatant was filtered through 0.45 µm cut-off polyethersulphone filters or glass fibre.

An equal volume of cobalt-resin wash buffer [50 mM phosphate (Na^+^), pH 7.0, containing 300 mM NaCl] was mixed with the extract, and then the mixture was applied on to a HisPur^TM^ Co^2+^ gravity-flow column (1 ml bed volume) which was pre-equilibrated as in the “HisPur^TM^ Cobalt Resin protocol” (Thermo Fisher Scientific).

The flowthrough solution was collected and re-applied to the column, maximising the binding of His-tagged protein; non-specifically bound proteins were removed with wash buffer (2 ml). Elution buffers 1–4, comprising 10, 30, 90 or 270 mM imidazole [prepared by diluting 1.0 M imidazole (pre-adjusted to pH 7.0 with concentrated HCl) into wash buffer], were subsequently applied (2 ml each) to the column as for the wash buffer.

Eluents were dialysed (12–14 kDa cut-off, Medicell Membranes Ltd., London, U.K.) against water at 4°C for 1 day (three water changes), followed by freeze-drying. Protein was re-dissolved in a small volume of water and quantified by *A*_280_. Desired concentrations were adjusted by addition of water.

##### Verification of His_6_-tagged CUS1 heterologous expression

SDS–polyacrylamide gel electrophoresis (PAGE) was performed as described by [54] in a 1.5 mm-thick 15% gel. Gels were stained with 0.006% Coomassie Brilliant Blue R-250 (in 10% acetic acid) overnight, and then the background staining was removed in the same solvent (Supplementary Figure S6a).

For immunodetection, a nitrocellulose membrane was used according to the “iBlot 2 NC Transfer Stacks” protocol [https://www.thermofisher.com/document-connect/document-connect.html?url=https%3A%2F%2Fassets.thermofisher.com%2FTFS-Assets%2FLSG%2Fmanuals%2FMAN0009112_iBlot2DryBlotSystem_UG.pdf&title=VXNlciBHdWlkZTogaUJsb3QgMiBEcnkgQmxvdHRpbmcgU3lzdGVt]. The membrane was then blocked as described by [55]. Monoclonal anti-polyhistidine–HRP antibody (Alpha Diagnostic International, San Antonio, U.S.A.) was diluted (1 : 1500) in PBS containing 0.1% (v/v) TWEEN-20, and incubated with the membrane overnight at 4°C. Un-bound antibody was washed off with the same buffer numerous times at 4°C. “SuperSignal West Dura Extended Duration Substrate” (equal volumes of the commercial luminol and H_2_O_2_ solutions; Thermo) was then applied on to the membrane for 1 min, the membrane was covered with polythene film, placed in a cassette, and exposed to X-ray film (Kodak, Rochester, U.S.A.) for 1 s (Supplementary Figure S6b). Non-infiltrated *N. benthamiana* leaves (Supplementary Figure S6c,d) were also investigated as above.

## Results

### An insoluble cutin-like polymer becomes esterified to a soluble cutin acid by an epidermal transacylase activity *in situ*

To look for the proposed CCT reaction within the cuticle, we devised and optimised a sensitive *in-situ* radiochemical assay comparable to the XET assay that is commonly used for detecting transglycosylation reactions [46]. The assay differs fundamentally from previous transacylase assays used for studying *de-novo* cutin synthesis, which require “activated” donor substrates — an HFA-glycerol [13] or an HFA-CoA [56]. Our strategy relies on the postulated CCT's ability to use a small, ^3^H-labelled acceptor substrate plus a large, non-radioactive donor, to produce a large, radiolabelled product (Figure 1b). As the small acceptor for CCT assays we chose the widespread cutin component, HHA [14,17], which is soluble in aqueous and methanolic solvents. Its hydroxy group theoretically enables it to serve as an acceptor substrate to which a carboxy group from a donor substrate molecule could be attached via an ester bond. The assay relied on endogenous non-radioactive cutin as the large donor, which is insoluble in neutral and acidic solvents. The radiolabelled transacylation product would be large enough to be insoluble if the ester bond cleaved in the donor was near the carboxy-terminus (as shown in Figure 1b).

Experimental plant materials were selected [*Pisum sativum* L. (pea) epicotyl, *Solanum lycopersicum* L. (tomato) fruit and *Hylotelephium spectabile* (Boreau) H. Ohba (ice plant) leaf] on the basis that they have a thick epidermis, include three different aerial organs, and cover three phylogenetic orders (Fabales, Asterales and Saxifragales; representing rosids, asterids and “rosid-allies’, respectively).

Prior to the *in-situ* experiments, we deliberately membrane-damaged the native epidermal samples, and adequately washed them in buffer, to remove ATP, CoA and glycerol, which might otherwise have produced acyl donors ([^3^H]HHA-CoA or even [^3^H]HHA-glycerol) that are involved in *de-novo* cutin synthesis.

**Figure 3.**
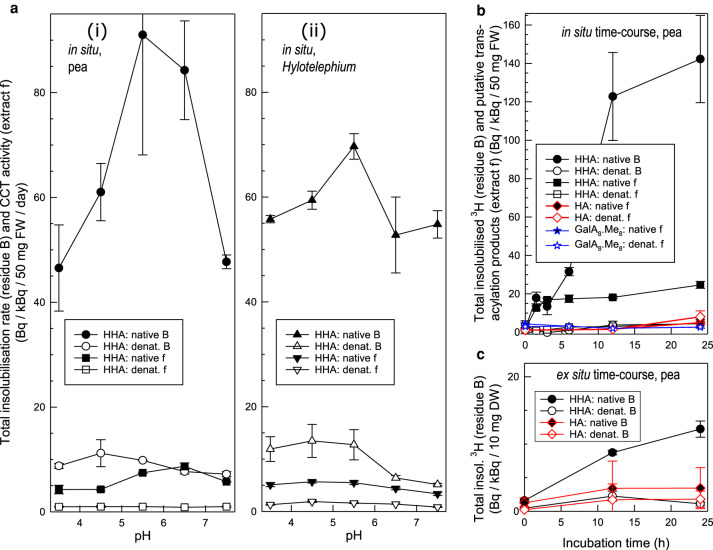
Effect of pH and incubation time on the incorporation of radiolabelled substrates into epidermis *in situ* and *ex situ*. (**a**) Effect of pH on putative CCT activity *in situ* in (i) pea epicotyl and (ii) *Hylotelephium* leaf epidermis. Each native or heat-denatured, blot-dried, 50-mg epidermis sample was incubated with 0.32–0.62 kBq [^3^H]HHA in 300 µl buffer at 20°C for 24 h. Total methanol-insoluble ^3^H-products (residue B of Figure 2) were assayed. The material solubilised by alkaline hydrolysis (extract f of Figure 2; putative ester-bonded material) was separately assayed. Filled symbols, native epidermis; open symbols, heat-denatured. Bars indicate standard errors (*n *= 3). (**b**) Time-courses of *in-situ* incorporation of [^3^H]HHA, [^14^C]hexadecanoic acid (HA) and Me_8_-[^3^H]GalA_8_-ol (methylesterified oligogalacturonide) by pea epicotyl epidermis at pH 5.5. Methods as in (**a**), except that the exogenous substrate was 0.10 or 0.13 kBq [^3^H]HHA, 0.24 kBq [^14^C]HA or 0.42 kBq Me_8_-[^3^H]GalA_8_-ol. Total ^3^H-labelled products insoluble in acidified methanol (residue B of Figure 2), and material subsequently alkali-solubilised (extract f; putative ester-bonded material), were assayed for radioactivity. Filled symbols, native epidermis; open symbols, heat-denatured. Data from three independent experiments (±SE, *n* = 3). (**c**) Time-course of *ex-situ* [^3^H]HHA and [^14^C]HA incorporation into the total methanol-insoluble fraction of heat-denatured pea epicotyl epidermis (10 mg), catalysed at pH 5.5 by exogenous enzymes extracted from non-denatured pea epicotyl epidermis. Acceptor substrate, 0.64 kBq [^3^H]HHA or 0.25 kBq [^14^C]HA. Filled symbols, native protein extract; open symbols, heat-denatured. Bars indicate standard errors (*n* = 3).

In *in-situ* experiments on pea epicotyl and *Hylotelephium* leaf epidermis, radioactivity was indeed incorporated from [^3^H]HHA within 24 h into material that was insoluble in acidified methanol (residue B of Figure 2) (Figure 3a). A proportion of this material remained insoluble in three further neutral solvents and proteinase K, yielding residue F. Analysis of the intermediary fractions will be discussed later, but primarily we focus on residue F. Cold alkali treatment of residue F was of particular interest as it was able to solubilise additional ^3^H (extract f of Figure 2) (Figure 3a), indicating attachment of an epidermal polymer to the [^3^H]HHA via an ester bond. This result thus suggests the existence of CCT activity. In this experiment, extract f accounted for ∼10% of the total ^3^H in residue B. Incorporation was maximal in the apoplastic pH range (4.5–6.5), and the reaction was strongly diminished if the epidermis was heat-denatured (Figure 3a; *P* < 0.05), compatible with the involvement of an apoplastic enzyme, such enzymes often being relatively heat-stable [57]. Such a pH optimum is close to that of the apoplastic CUS1 [58], but appreciably lower than that of the system reported by [56], which was dependent on ATP, CoA and a protoplasmic HFA-CoA synthetase.

At pH 5.5, *Hylotelephium* leaf and pea epicotyl epidermis gave higher total methanol-insoluble ^3^H (incorporation into residue B) than did tomato fruit epidermis (Supplementary Figure S1). We therefore selected pea epicotyls for more detailed study because they are quickly and easily grown.

Tritium incorporation from [^3^H]HHA into native pea epidermis was time-dependent (Figure 3b; *P*_native *vs.* denatured_ < 0.05). The yield of total insolubilised ^3^H-products (residue B) increased over the first 12 h then plateaued, whereas the plateau of ester-linked material (extract f) was reached by 3 h (Figure 3b). We suggest that the CCT enzyme was gradually denatured within ∼3 h, whereas other activities capable of generating neutral-solvent-soluble products (found in extracts b–e) persisted for 12 h. In *Hylotelephium* leaves, the incorporation of ^3^H persisted for at least 24 h (Supplementary Figure S2).

The next question was which functional group (–OH or –COOH) of HHA is involved in the transacylation reaction. It is more likely that a new ester bond would be formed to HHA's –OH group from an endogenous polymeric acyl group (e.g. existing cutin). The –COOH group of free HHA is not “activated” in any way and is unlikely to become activated in the frozen/thawed/washed epidermis, depleted of metabolites such as CoA and ATP. Support for a key role of the –OH group was provided by the observation that [^14^C]HA, which has no –OH group, was not incorporated in our *in-situ* system (Figure 3b; *P*_native *vs.* denatured_ > 0.05). Other “control” compounds, such as [^3^H]HHA derivatives with the –OH group blocked (e.g. *O*-acetyl-[^3^H]HHA), were not included here because [^14^C]HA is more structurally similar to [^3^H]HHA, and the additional groups might sterically hinder the access of CCT enzyme to the substrate. Furthermore, endogenous enzymes (e.g. acetylesterases) might remove the blocking group, resulting in false-positive results.

### Cutin-like polymer esterified to soluble cutin fragment by extractable epidermal transacylase activity *ex situ*

Next, we attempted to solubilise CCT activity from native pea epicotyl epidermis. Extracted proteins proved able to catalyse the time-dependent incorporation of radioactivity from [^3^H]HHA into epidermal walls (including cuticle) whose own enzymes had been heat-denatured (Figure 3c). Such incorporation was dependent on the added proteins and did not occur if these proteins were heat-denatured, supporting the existence of an extractable transacylase. The lower total incorporation in *ex-situ* (Figure 3c) than in *in-situ* assays (Figure 3b) may be because the transacylase was not readily water-extractable (possibly embedded in the hydrophobic cuticle) or it was partially denatured during extraction. As in *in-situ* assays, the ability of [^14^C]HA to be incorporated into epidermis was also assayed, as a test of the capability of the ex-situ system in esterifying –COOH groups. Consistently, [^14^C]HA was not incorporated (Figure 3b), strongly suggesting that intracellular long-chain acyl-CoA synthetases and acyl-glycerol synthases [24,56,59] did not produce “high-energy” radioactive acyl donors (e.g. [^3^H]HHA-CoA or [^3^H]HHA-glycerol) *de novo*, that could have introduced false-positive results. Taken together, the newly developed *in-situ* and *ex-situ* assays with [^3^H]HHA suggest the existence of a transacylase activity (CCT) that can use an insoluble endogenous polymer possessing ester bonds (e.g. cutin) as donor substrate and the soluble cutin-acid [^3^H]HHA as the acyl acceptor. The nature of the acyl donor is further investigated in the following section.

### Endogenous cutin forms ester bonds with exogenous [^3^H]HHA *in situ*

To explore further the transacylation reaction, we employed chemical degradation strategies to investigate whether the [^3^H]HHA was incorporated intact and whether the endogenous polymeric donor substrate was cutin.

### Polymers become ester-bonded to intact [^3^H]HHA, and low-M_r_ by-products are also formed

**Figure 4.**
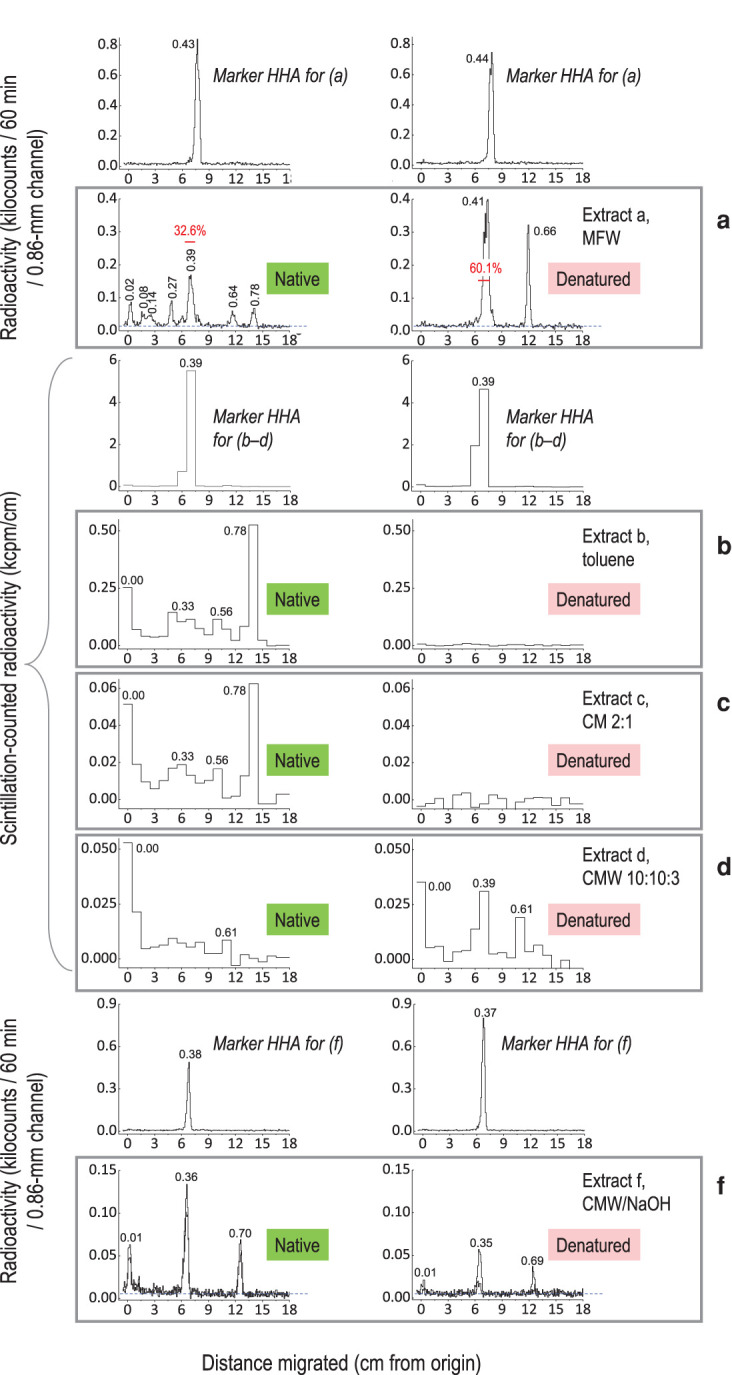
Chromatographic analysis of enzymic products from pea epicotyl *in-situ* assays with [^3^H]HHA. Blot-dried pea epicotyl epidermis (200 mg, cutin donor source; native or heat-denatured) was incubated with 12.1 kBq [^3^H]HHA in 1.2 ml buffer (pH 5.5) for 24 h. The plots show TLCs of reaction products solubilised from the epidermis by, sequentially extractants **a**, acidified methanol; **b**, toluene; **c**, chloroform/methanol (CM, 2 : 1); **d**, chloroform/methanol/water (CMW, 10 : 10 : 3); **f**, CMNaOH (CMW containing 0.52 M sodium hydroxide). Between **d** and **f**, the insoluble material was digested with proteinase, which, however, released negligible ^3^H. Replicate TLCs were performed for (**f**) and the two profiles are superimposed. Each ‘marker’ profile (∼1 kBq [^3^H]HHA) refers to the sample(s) shown below it. TLC was in toluene/acetic acid, 9 : 1, with three ascents. Figures given above peaks in black are *R*_F_ values. Figures given in red report the ^3^H in the HHA peak (red line) as a percentage of total ^3^H in the profile (corrected for the background, which is shown as a dashed blue line; 13 counts/60 min/channel in Figure 4a).

The above data show that the apparent ester-bonding of insoluble polymers to exogenous [^3^H]HHA (CCT products in extract f) accounted for ∼1–3% of the total supplied ^3^H. Concurrent reactions forming low-M_r_ by-products would compete with the CCT reaction. We therefore investigated chromatographically the material not in extract f (i.e. solubilised in extractants a–d; Figure 2, Table 1), to look for such by-products. Products extractable in acidified methanol (extract a) were expected to include unchanged [^3^H]HHA plus any small, polar metabolites; products subsequently extractable from residue B in non-polar solvents (extracts b–d; Table 1) may have included non-polar products. By-products, formed *in situ* within 24 h by native and heat-denatured pea epicotyl epidermis, are shown in Figure 4. About 33% of the ^3^H remained as [^3^H]HHA (Figure 4a, left profile). One of the by-products (*R*_F_ ∼ 0.65) was not an enzymic product, as it was also formed in the presence of heat-denatured epidermis (Figure 4a, right profile) and in buffer alone during the SpeedVac-drying process (Supplementary Figure S3). This substance appears to be [^3^H]OHA (16-oxo[16-^3^H]hexadecanoic acid), which would be produced, together with radioactive water, by oxidation of [16-^3^H]HHA (represented below as R–CH**T**OH, where **T** is tritium):

$$2\text{HOOC}\text{-}(\text{C}{\text{H}}_{2}{)}_{14}\text{-}\text{CH}T\text{OH}+2[\text{O}]\to \text{HOOC}\text{-}(\text{C}{\text{H}}_{2}{)}_{14}\text{-}\text{C}T\text{O}+\text{HOOC}\text{-}(\text{C}{\text{H}}_{2}{)}_{14}\text{-}\text{CHO}+\text{HTO}+{\text{H}}_{2}\text{O}$$

(where [O] is an oxidant, possibly O_2_ or a reactive oxygen species). OHA had been used as an intermediate in the preparation of [^3^H]HHA and thus its *R*_F_ value was known (Supplementary Figure S4a). The other low-M_r_ by-products, evidently formed by epidermal enzymes (Figure 4a, left profile), remain unidentified: potentially they include small oligo-cutin and/or wax esters.

**Table 1:** Quantification of products formed *in situ* from [^3^H]HHA by pea epicotyl epidermis

Extractant (a–g′) or residue	% of supplied radioactivity ± SE	
	Native epidermis	Heat-denatured epidermis
a. Acidified aqueous methanol^1^ (methanol/formic acid/water) (9 : 1 : 1) (MFW) *in vacuo*	55.2 ± 4.6	92.2 ± 1.1
b. Toluene	7.5 ± 1.6**	0.5 ± 0.3
c. Chloroform/methanol (2 : 1) (CM)	2.3 ± 0.5*	0.2 ± 0.1
d. Chloroform/methanol/water (10 : 10 : 3) (CMW)	0.9 ± 0.6	0.0 ± 0.0
e. Proteinase K (releasing putative peptide-bonded material)	0.4 ± 0.1	0.4 ± 0.2
f. Chloroform/methanol/NaOH (CMNaOH) (releasing putative ester-bonded material)	1.9 ± 0.4**	**0.4 **±** 0.2**
g. XEG (releasing putative xyloglucan-bonded material)	0.0^2^	0.0^2^
g′. 2M TFA (releasing putative unspecified matrix polysaccharide-bonded material)	0.2 ± 0.2	0.0 ± 0.0
Final insoluble residue (H or H′)	0.0 ± 0.0	0.0 ± 0.0
Total	68.4	93.7

In three independent experiments, blot-dried pea epicotyl epidermis (200 mg; native or heat-denatured) was incubated with 2.81, 4.28 or 12.1 kBq [^3^H]HHA in 300 µl buffer (pH 5.5) at 20°C for 24 h. The radiolabelled products were then fractionated as in Figure 2. Errors are given as ± SE (*n *= 3 biological replicates).

* *P*_native vs. denatured_ < 0.05. ** *P*_native vs. denatured_ < 0.01.

1 Extract ‘a’ includes only the solutes in the first MFW extraction, performed in vials. The epidermis was subsequently washed more thoroughly by irrigation with MFW by a paper chromatography setup, where the chromatographically mobile radioactivity was not assayed. This explains why the total is <100%;

2 No SE given (*n* = 1).

Three subsequently applied neutral solvents (b–d; toluene, CM and CMW) solubilised additional by-products from the [^3^H]HHA-incubated native epidermis (Figure 4b–d; Table 1). Formation of these products was largely enzymic as they were not (or were significantly less) produced by denatured epidermis.

Despite the formation of low-M_r_ by-products, we obtained evidence that the polymeric ester-linked ^3^H (in residue F) had been incorporated largely in the form of intact [^3^H]HHA moieties. Alkaline CMW (extractant f) solubilised a substantial amount (1.9% of the fed [^3^H]HHA in this experiment) even though thorough washing with neutral CMW had solubilised only 0.9% of the fed [^3^H]HHA (Table 1). This alkali-solubilised ^3^H, thus deduced to have been ester-linked within the epidermis, mainly co-migrated with authentic HHA (Figure 4f) and had thus been ester-bonded intact. A minority co-migrated with [^3^H]OHA, and a further proportion was chromatographically immobile (unidentified).

### Ester bonds and only ester bonds

Although alkali (extractant f) released ^3^H-labelled material from the covalently bound *in-situ* products of [^3^H]HHA (Figure 3; Table 1), interestingly, negligible radioactivity was left insoluble after alkaline hydrolysis (Table 1). [^3^H]HHA molecules held by protein–[^3^H]HHA peptide or isopeptide bonds [60] or polysaccharide–[^3^H]HHA glycosidic bonds [61] would have been stable to cold alkali and thus present in residue G (Figure 2), but would have released the ^3^H into hot trifluoroacetic acid (TFA; extractant g′), which hydrolyses amide and glycosidic bonds. In addition, to look specifically for xyloglucan that may have become glycosidically bonded to [^3^H]HHA, we digested other samples of the alkali-stable residue G with xyloglucan endoglucanase (XEG, which hydrolyses the backbone of xyloglucan; extractant g; Figure 2). Little or no radioactivity was solubilised by extractants g′ or g (Figure 2, Table 1), indicating negligible polysaccharide–HHA bonding. Nor was there any radioactivity in the final pellet (Table 1), precluding any possibly acid-stable cellulose–[^3^H]HHA linkages. Furthermore, [^3^H]HHA molecules held by cutan (a biopolymer mainly composed of alkanes and alkenes and sometimes co-occurring with cutin in plants) [62,63] via ether or carbon–carbon bonds would have resulted in ^3^H being found in the final insoluble residue (H or H´; Figure 2). The findings (Table 1) therefore highlight that esterification was the only appreciable mechanism by which the exogenous [^3^H]HHA molecules became covalently associated with endogenous polymers, agreeing with our hypothesis of a CCT activity.

### Evidence that the polymeric donor substrate is cutin

The endogenous high-M_r_ donor substrate's “activated” carboxy group, which became ester-bonded to the –OH of [^3^H]HHA, could itself have been in cutin (ester linkage: R-COOR′) as we hypothesised. But alternatively, some other acyl linkage such as a peptide or isopeptide bond (R-CONHR′; e.g. in the backbone of a protein or in a glutamyl-polyamine side-chain [64]) or a primary amide (R-CONH_2_, e.g. the side-chain of asparagine or glutamine) are also possible.

To test for possible protein–[^3^H]HHA ester bonds as *in-situ* products, we digested epidermal samples (residue E) with proteinase K. Little ^3^H was solubilised by proteinase (extract e; Figure 2, Table 1), indicating that proteins were not the major acyl donors for the detected transacylase activity. Furthermore, the small amount of ^3^H solubilised by proteinase did not differ between native and denatured epidermis, indicating that any protein–[^3^H]HHA attachment had not been brought about enzymically.

Besides cutin, another possible high-M_r_ ester-type donor substrate could have been methyl-esterified pectin. As a model substrate to explore this, we synthesised fully methyl-esterified [^3^H]GalA_8_-ol (reductively tritiated octasaccharide of homogalacturonan; Supplementary Figure S5). In *in-situ* experiments, no radioactivity was incorporated from Me_8_-[^3^H]GalA_8_-ol into pea epicotyl epidermal walls (Figure 3b), so there was no evidence for a transacylase capable of using methyl-esterified pectin as donor and any insoluble endogenous alcohol (cutin or other) as acceptor substrate under our experimental conditions, despite the fact that cutin is suggested to interact closely with methyl-esterified galacturonan [65].

In conclusion, out of the three plausible types of insoluble high-M_r_ acyl-donor substrate (cutin, pectin and protein) in our *in-situ* transacylase assay, we have evidence against pectin and protein, and we therefore favour cutin as the most likely acyl donor.

### An *endo*-transacylase

Cleaving an endogenous high-M_r_ cutin molecule at the ester bond of a hydroxy-terminal fatty acid residue followed by ester-bonding of this residue to the –OH group of exogenous [^3^H]HHA by transacylation would yield a radioactive dimer, HFA–[^3^H]HHA, which would be soluble in methanol. It is possible that some of the unidentified products seen in Figure 4a (native) include HFA–[^3^H]HHA dimers or similar substances. However, the observation that the radioactive products of interest were big enough to be methanol-insoluble (Table 1) shows that the cutin donor substrate was cleaved at an ester bond towards the middle of the polymer or close to its carboxy-terminus. We conclude that the enzyme activity which yields a radioactive product appearing in extractant f is an *endo*-CCT.

### CCT activity correlates with the growth rate

If CCT activity is involved in loosening the cuticle, facilitating organ expansion, we would predict higher activity in young, rapidly growing organs. The effect of age on *in-situ* CCT activity was therefore tested in all three plant species.

**Figure 5.**
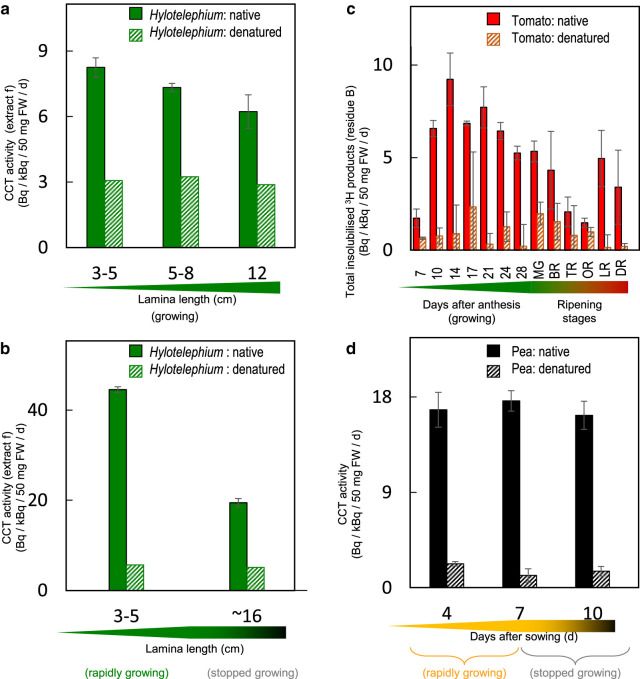
Effects of plant growth and development on CCT activity *in situ*. Each *in-situ* assay was performed on 50 mg of native or heat-denatured, blot-dried epidermis, with 0.14–0.75 kBq [^3^H]HHA in 300 µl pH 5.5 buffer at 20°C for 24 h. In a, **b** and **d**, the CCT products (ester-bonded radioactivity detected in extract f; Figure 2) were assayed. In **c**, total methanol-insolubilised products (residue B; Figure 2) are reported. (**a**) Epidermis samples from differently aged, expanding *Hylotelephium* leaves; 0.72 kBq [^3^H]HHA. Bars indicate standard errors (*n *= 3). (**b**) Epidermis samples from expanding versus fully expanded *Hylotelephium* leaves; 0.15 kBq [^3^H]HHA. Bars indicate range (*n *= 2). (**c**) Epidermis samples from tomato fruit (cv. Ailsa Craig) at different developmental stages; 0.14 kBq [^3^H]HHA. Bars indicate range (*n *= 2). At 7–28 days after anthesis, the fruit are expanding; thereafter they are described [90] as MG, mature green (growth stopped); BR, breaker (10% colour change); TR, turning (30% colour change), OR, orange; LR, light red; DR, deep red. (**d**) Epidermis samples from etiolated pea epicotyls; 0.69 kBq [^3^H]HHA. Bars indicate standard errors (*n *= 3).

In *Hylotelephium* leaf epidermis, there was a trend towards lower *in-situ* CCT activity (per mg of epidermis) with increasing lamina length, and thus age, within the range 3–12 cm (Figure 5a). In an independent experiment, the activity diminished in 16 cm (fully expanded) laminae more than two-fold (Figure 5b). In tomato fruit epidermis, there was a transient peak in the formation of methanol-insoluble products at ∼14–21 days after anthesis (DAA) (Figure 5c), during which time the fruit grew from ∼1.5 to ∼2.5 cm diameter. Interestingly, a second peak occurred in the red ripening stages after the fruits had reached their maximum diameter of ∼3.8 cm. Etiolated pea epicotyls consistently exhibited high *in-situ* CCT activity, and there was no appreciable change between 4 and 10 days after sowing (Figure 5d); maximum epicotyl length (∼16 cm) had been reached by ∼7 days, and negligible further elongation occurred between 7 and 10 days in the dark.

Thus, at least in tomato and *Hylotelephium*, CCT activity correlated positively with the organ growth rate. In pea seedlings, it is possible that beyond 7 days in the dark, other factors than CCT activity (e.g. energy supply and thus turgor pressure) were limiting epicotyl growth.

### CCT activity is not attributable to cutin synthase

**Figure 6.**
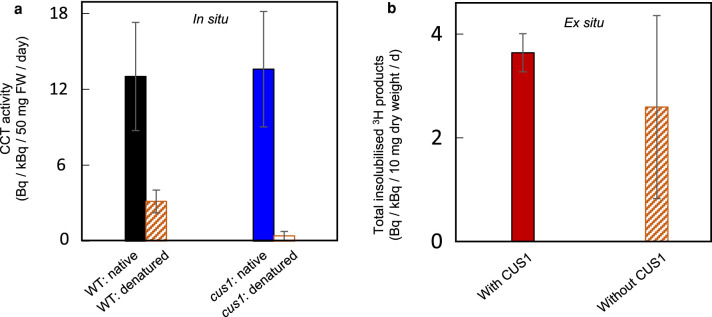
Test of CUS1's CCT activity *in situ* and *ex situ*. (**a**) Comparison CCT activity (*in-situ* formation of ester-bonded [^3^H]HHA; extract f of Figure 2) in epidermis of wild-type tomato (cv. M82) and the isogenic *cus1* mutant (diminished in cutin synthase) at pH 5.5. Native or heat-denatured, blot-dried epidermis (50 mg) was incubated with 0.16 or 0.92 kBq [^3^H]HHA in 300 µl buffer at 20°C for 24 h. Bars indicate range (*n *= 2) of two independent experiments. (**b**) Testing whether *N. benthamiana*-produced CUS1 catalyses [^3^H]HHA incorporation into the total methanol-insoluble fraction (residue B) of heat-denatured pea epicotyl epidermis (10 mg) *ex situ* at pH 5.5. Epidermis samples were incubated with 1.03, 1.16 or 1.38 kBq [^3^H]HHA with or without the cobalt-purified His_6_-tagged CUS1 (1 µg) in 300 µl buffer at 20°C for 1 day. Bars indicate standard errors (*n *= 3; results from three independent experiments).

The only well-studied cutin transacylase in the apoplast, tomato CUS1 [13,26,66], has a pH optimum similar to that of the observed CCT activity (Figure 3a). As a GDSL protein [67], CUS1 may be promiscuous with respect to its substrate specificity. We therefore investigated CUS1 as a candidate possibly responsible for CCT activity despite the non-involvement of CUS1's only known donor substrates (HFA-glycerols) [13,66] in the CCT assay. In *in-situ* assays, *cus1* mutant tomato fruit epidermis exhibited as much CCT activity as the wild-type (cv. M82) (Figure 6a), showing that CUS1 is not responsible for the majority of the observed CCT activity. Furthermore, in *ex-situ* assays, purified His_6_-tagged tomato CUS1, heterologously produced in *N. benthamiana* (Supplementary Figure S6a–d; and evidently active as it was able to catalyse fatty-acyl ester hydrolysis; Supplementary Figure S6e), did not exhibit CCT activity on the cutin of denatured pea epidermal walls (Figure 6b; *P*_with *vs.* without CUS1_ > 0.05). Therefore, the observed CCT activity is not due to tomato CUS1or its pea and *Hylotelephium* equivalents.

### Potential effectors on CCT activity

Some bacterial GDSLs are activated by metal cofactors — e.g. the esterase estSL3 of *Alkalibacterium* by 1 mM Ca^2+^, K^+^, Ni^2+^ and Mg^2+^ [68]. We therefore tested several metal ions at 10 mM on CCT activity *ex situ* (Table 2). Ca^2+^ appeared to enhance CCT activity by 25% but the effect was not significant. The other metal ions tested had little stimulatory effect; Zn^2+^ and Co^2+^ were moderately inhibitory. The chelating agent, EDTA, also had little effect, supporting the conclusion that the protein responsible for CCT activity did not depend on divalent metal ions.

**Table 2:** Effects of additives on CCT activity *ex situ*

Added ions	Relative ^3^H incorporation (% of control ± range)
Control a	100 ± 5.4
10 mM Ca^2+^	125 ± 21
10 mM Fe^2+^	101 ± 8
10 mM Mg^2+^	90.4 ± 9.2
10 mM Ni^2+^	89.5 ± 15.0
10 mM Zn^2+^	69.4 ± 5.4*
10 mM K^+^	94.4 ± 29.1
10 mM Co^2+^	66.1 ± 3.2**
10 mM EDTA	108 ± 18
Control b	100 ± 8.0
10 mM dithiothreitol	91.1 ± 10.4

In two independent experiments, an enzyme extract [prepared in 350 mM succinate (Na^+^), pH 5.5, with 1% v/v Triton X-100 and 1% w/v polyvinylpolypyrrolidone] from pea epicotyl epidermis was added to 10 mg heat-denatured and thoroughly dried pea epidermis with 0.36–0.62 kBq [^3^H]HHA plus the ions listed. After 24 h at 20°C, ^3^H incorporated into ester-linked CCT reaction products (equivalent to extract f of Figure 2) was recorded. Data show mean ± range (*n* = 2 biological replicates). * *P* < 0.05; ** *P* < 0.01.

CCT activity in *ex-situ* assays was also unaffected by the sulphydryl reagent, dithiothreitol (Table 2), which would minimise disulfide bridging of cysteine residues. It has been hypothesised that CUS1 contains three disulfide bonds based on *in-silico* prediction (DiANNA, http://clavius.bc.edu/∼clotelab/DiANNA/) [69], and many other GDSLs are also thought to possess multiple disulfides. However, if CCT activity does not depend on disulfide bonds (Table 2), it may lack S–S bonds; or, if present, they may not affect the enzymic activity.

## Discussion

### Discovery of CCT activity via radiochemical assays

Numerous independent experiments, involving *in-situ* and *ex-situ* assays on all three plant species and organs tested, demonstrated that exogenous [^3^H]HHA (a soluble, radiolabelled cutin-acid; the acyl acceptor in Figure 1b) can indeed be enzymically incorporated into the plant epidermis (e.g. Figure 3). The ^3^H-labelled product formed was insoluble in all neutral and acidic solvents tested, indicating that the [^3^H]HHA had become anchored to a high-molecular-weight component of the epidermal wall. Cold alkali subsequently released the radioactivity in the form of HHA, indicating that the latter had been immobilised as an intact unit via an ester bond. If the [^3^H]HHA had been immobilised via peptide, glycosidic, ether or C–C bonds, it would not have been released by cold alkali (which was applied in chloroform/methanol containing only 13% water, thus not an effective solvent for proteins and polysaccharides). Non-hydroxylated [^14^C]HA was not incorporated, pointing to the –OH group of HHA as the site through which an ester bond was formed. Thus, as predicted (Figure 1b), the HHA was the acceptor substrate of a transacylation reaction.

The insoluble, high-molecular-weight acyl donor substrate implicated in the observed reaction could a priori be cutin or pectin (some of whose carboxy groups are “activated” as methyl esters) or proteins (some of whose carboxy groups are “activated” as amides). To test for the possible contribution of pectic transacylation as an alternative explanation of our observed [^3^H]HHA bonding, we conducted *in-situ* experiments with an [^3^H]oligogalacturonide methyl-ester (Figure 3b), relying on endogenous epidermal polymers as potential acceptor substrates in reactions of the type

$$\text{Gal}{\text{A}}_{8}\text{-}\text{ol}.\mathrm{OM}{\text{e}}_{8}+\text{HO}\text{-}\text{R}\to \text{Gal}{\text{A}}_{8}\text{-}\text{ol}.\mathrm{OM}{\text{e}}_{7}\text{-}\text{O}\text{-}\text{R}+\text{HO}\text{-}\text{Me}$$

where R is an unspecified epidermal polymer such as cutin and HO–Me is methanol. Such incubations did not generate ester bonds. This observation makes it highly unlikely that, in our incubations (e.g. Figure 3), endogenous pectin could have participated as the donor substrate in apoplastic transesterification reactions with [^3^H]HHA as acceptor substrate, such as

$$(\text{Gal}{\text{A}}_{n}.\mathrm{OM}{\text{e}}_{m})+\text{HO}\text{-}\text{hexadecanoate}\to (\text{Gal}{\text{A}}_{n}.\text{OM}{\text{e}}_{m_{\text{-}1}})\text{-}\text{O}\text{-}\text{hexadecanoate}+\text{HO}\text{-}\text{Me}$$

where (GalA*_n_*.OMe*_m_*) is a methyl-esterified, high-molecular-weight cell-wall pectin.

Furthermore, pectins (or any other polymers) could not be the acceptor substrates involved in ester-bonding of HHA to insoluble components because the supplied [^3^H]HHA was not “activated” and would not have become activated in our assays because of the lack of ATP and CoA. This theory is supported by the fact that [^14^C]HA could not be incorporated into epidermis (Figure 3b,c), as it lacks of the –OH group and its –COOH group was not activated. In summary, despite the long-standing suggestion of covalent attachment of pectin to cutin [14,65], we did not observe pectin–HHA ester-bonding in our experimental protocols.

Moreover, the failure of proteinase, XEG and TFA to solubilise [^3^H]HHA-containing fragments (Table 1) argues against peptide or polysaccharide donor substrates. Cutin is thus the most plausible of the three proposed polymeric donor substrates, a conclusion supported by the high abundance of cutin in epidermis.

The insolubility of the [^3^H]HHA-containing product in neutral and acidic extractants a–d (Figure 2) indicates that it had a high molecular weight, and thus that the transacylation event occurred on the donor cutin molecule relatively close to the carboxy-terminus. We conclude that the enzyme involved is an *endo*-transacylase (or even one that cleaves the cutin at the last ester bond, adjacent to the carboxy-terminus), capable of transferring a large segment of insoluble cutin onto the –OH group of the exogenous [^3^H]HHA. If it were an exo-transacylase, cleaving cutin at the ester bond of a hydroxy-terminal HFA residue, the radiolabelled product would have been a dimer (HFA–[^3^H]HHA), which would be soluble in acidified methanol (extractant a). Such an exo-enzyme may also exist, in addition to the reported endo-enzyme, possibly yielding some of the unidentified radioactive products seen in Figure 4a (left panel); but this hypothesis would require further experimentation.

The relatively low proportion of exogenous [^3^H]HHA incorporated into cutin (∼1.5% when corrected for the denatured control; Table 1) under our conditions could be because HHA (as a C_16_ mono- rather than di-hydroxy fatty acid) is not the optimum substrate for CCT activity, especially in pea, whose cutin is dominated by C_18_ HFAs [70]. The difference between the quantities of total insolubilised ^3^H (residue B) and extract f (deemed to be CCT products) shows that some of the [^3^H]HHA had become associated with the epidermis via non-covalent bonds. We confirmed (Table 1) that the neutral extractants (b–e) were capable of liberating this ^3^H from the epidermis, as expected for certain types of non-covalent bond. Its inextractability in acidified aqueous methanol but extractability in toluene etc. suggests that the ^3^H solubilised by extractants b–e had been converted to highly non-polar metabolites or become trapped within an oily or waxy component of the epidermis. Such trapping of enzymic products is also revealed in the 68% recovery of fed radioactivity from native epidermal samples, compared with 94% from the control (Table 1). Table 1 also shows that, of the material trapped in residue B, 85% was releasable by neutral extractants (b–e), and 13% came out in extract f; this calculation is corrected for the small amounts of ^3^H trapped by heat-denatured epidermis.

Our data thus indicate the existence of a cutin:cutin endo-transacylase (endo-CCT) activity in the epidermis of aerial organs of pea, tomato and *Hylotelephium*, capable of using a monomeric cutin-acid as the acceptor substrate. It is likely that *in vivo* in the absence of exogenous substrates, the main acceptor substrate would be polymeric (probably reticulated) cutin rather than a monomer, meaning that both substrate and product are insoluble. Such an activity could transiently loosen the cuticle, facilitating expansion or other restructuring-dependent events in the epidermis and thus of the whole organ.

Our finding provokes the question of how plants orchestrate the symphony of different known cell-wall re-modelling mechanisms, including transglycanases [47,48],transglycosidases [71], glycanases [72], glycosidases [73], expansins [74], peroxidases [75], hydroxyl radicals [76], pectate lyases [77], pectinesterases [78] and other esterases [79], and now a cutin-acting transacylase CCT — each potentially contributing to the control of plant growth.

### Towards the identification of the CCT protein(s)

Even though both CUS1 and the CCT activities peaked in tomato fruit at ∼15 DAA (Figure 5c) [13], our *in-situ* and *ex-situ* evidence indicates that tomato CCT activity is not attributable to CUS1, the only well-characterised cutin transacylase in the apoplast (Figure 6). However, their approximate co-expression in tomato fruit may suggest that they are both regulated by the same transcription factor, SHINE3 [80].

It is possible that CCT activity is attributable to a GDSL family transacylase other than CUS1. GDSLs are widespread from bacteria to mammals [67]. Arabidopsis has genes for 108 GDSLs, 99 of which have a signal peptide predicted to target the protein to the endoplasmic reticulum, Golgi or apoplast [81]; and apoplastic targeting has been confirmed in some cases [13,26,82]. Most plant GDSLs are of unknown function, but their postulated enzymic activities include hydrolysis (cutinase [25] and acetyl/butanoyl esterase [83]) and transacylation (donor substrate isobutyryl-glucose [84] or chlorogenic acid [82]). The expression of plant GDSLs is highly modulated by developmental cues and environmental stresses, especially drought, implying biological roles [85]. The finding that red light and jasmonate up-regulate a GDSL gene (*GER1*) in rice, coincidently with inhibiting coleoptile elongation [86], may suggest a role governing wall extension. Additional GDSL family transacylases other than CUS1 have been speculated to remodel cutin, potentially creating cutin–polysaccharide bonds [69]. Other GDSL proteins than CUS1, may thus potentially account for CCT activity.

Through *in-silico* analysis (http://tea.solgenomics.net/), we found that two un-investigated tomato genes encoding secreted GDSLs (locus names: *Solyc02g077330* and *Solyc12g017460*) are co-expressed with *CUS1* (*Solyc11g006250*) in the pericarp of 5- and 10-DAA M82 tomato fruits. These two GDSLs also share ∼30% amino acid identity with CUS1 (an aligned map is shown in Supplementary Figure S7), further suggesting that they may catalyse reactions of cutin metabolism. Nevertheless, whether they are regulated by SHINE3 and/or confer CCT activity remains to be answered.

Beyond the GDSL family, bodyguard (BDG) proteins as apoplastic enzymes with cutin-modifying activities [87] can also be considered as CCT candidates, especially as their expressions [45] and CCT activity (Figure 5c) have a second peak during tomato ripening. Numerous other a/b-fold proteins are known in plants (e.g. [85,87]), and may in future be surveyed for CCT activity.

In summary, our results exclude the only well-defined apoplastic cutin transacylase (CUS1) as a CCT candidate. Our assays and proposals may provide valuable information for future transcriptomic and proteomic studies to identify the CCT protein that remodels cutin.

### CCT's physiological function

We hypothesised that CCT activity is required during rapid organ growth to transiently loosen cutin since cutin has been proposed to limit cell expansion [41,45]. We explored this idea by testing the correlation between developmental stages and the activity. CCT activity was highest at the young, rapidly expanding stages of *Hylotelephium* leaf and tomato fruit development, followed by a gradual decrease with age and decelerating growth (Figure 5a–c), supporting the suggestion that CCT activity is involved in rapid organ expansion. The slightly acidic pH optimum (Figure 3a) may also point to a possible involvement in (acid) growth [88]. Most other wall-remodelling enzyme activities, e.g. XET, also have slightly acidic pH optima [46]. In tomato fruit, CCT activity was also observed to be up-regulated again after the cessation of cell expansion, probably paralleling the structural rearrangement of the cutin matrix during ripening [89].

Exploring whether diverse, epidermis-targeting growth regulators (e.g. auxin action on pea epicotyls) [39] stimulate CCT activity will contribute to solving CCT's physiological function(s).

## Conclusions

Based on discovery of the CCT reaction mechanism and its characteristics, we suggest that cutin can be re-structured by being transiently cleaved to enable epidermal loosening and then re-joined to restore cuticular strength (Figure 1a) in an expanded cuticle. We devised and optimised a portfolio of sensitive *in-situ* and *ex-situ* radiochemical assays by which to detect a cutin-acting transacylase activity (endo-CCT) in the plant shoot epidermis. For practical reasons, the activity which we detected *in vitro* was cutin:cutin-acid transacylation (cutin + [^3^H]HHA → cutin–[^3^H]HHA + cutin), though we suggest that the major *in-vivo* reaction catalysed is cutin:cutin transacylation (cutin + cutin → cutin + cutin), which would be difficult to detect. The developmental stage-dependent CCT activity together with its slightly acidic optimum pH value support the hypothesis that CCT serves a role in organ expansion — the epidermis generally being the limiting tissue for stem, leaf and fruit growth [40]. The protein responsible for CCT activity was not identified in this study, but our evidence indicates that it is not a side-reaction catalysed by the previously reported cutin synthase, CUS1. Our biochemical evidence provides a good starting point for further characterisation of CCT via proteomic and transcriptomic approaches. The methodology developed provides a robust and quantitative tool to probe cutin transacylases *in vivo* and *in vitro*, which will enable more detailed investigation of the occurrence and physiological roles of CCT.

## Data Availability

All data generated or analysed during this study are included in this published article (and its Supplementary Information Files).

## Open Access

Open access for this article was enabled by the participation of University of Edinburgh in an all-inclusive *Read & Publish* pilot with Portland Press and the Biochemical Society under a transformative agreement with JISC.

## Abbreviations

CCT: cutin:cutin transacylase
CM: chloroform/methanol
CMW: chloroform/methanol/water
HA: hexadecanoic acid
HFAs: hydroxy-fatty acids
HHA: 16-hydroxy-[^3^H]hexadecanoic acid
MFW: methanol/formic acid/water
OHA: 16-oxo[16-^3^H]hexadecanoic acid
PCC: pyridinium chlorochromate
QIAGEN: QIAquick Gel Extraction kit
TFA: trifluoroacetic acid
TLC: thin-layer chromatography
XEG: xyloglucan endoglucanase
XET: xyloglucan endotransglucosylase
