# Supplementary material for: Cutin:cutin-acid endo-transacylase (CCT), a cuticle-remodelling enzyme activity in the plant epidermis
Source: Biochem J. 2021 Feb 24;478(4):777–98. doi: 10.1042/BCJ20200835 (PMC7925011; doi:10.1042/BCJ20200835)
Supplement: Supplementary Figures S1-S7 [file BCJ-478-777-s1.pdf]

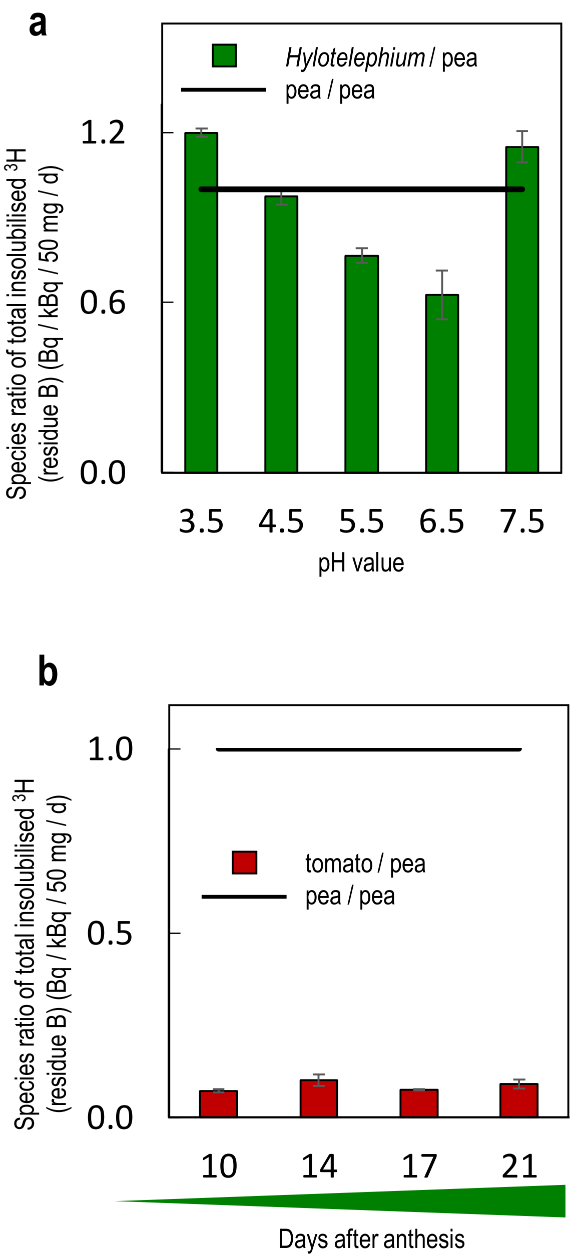

Fig. S1. Comparison of *in-situ* [^{3}H]HHA insolubilisation by epidermis of *Hylotelephium*, tomato and pea.

Blotted fresh epidermis (50 mg, from rapidly expanding organs) was incubated with 0.14–0.62 kBq [^{3}H]HHA in 300 µl buffer at 20°C for 24 h. Total ^{3}H that become insoluble in acidified methanol (= residue B of Fig. 2) was assayed.

(a) Comparison of epidermis from *Hylotelephium* leaf (adaxial) and pea epicotyl, at various pH values. Bars indicate standard errors (n = 3).

(b) Comparison of epidermis from tomato fruit (cv. Ailsa Craig) and pea epicotyl, at pH 5.5. Tomato fruits at 10–21 days after anthesis are rapidly expanding. Bars indicate range (n = 2).

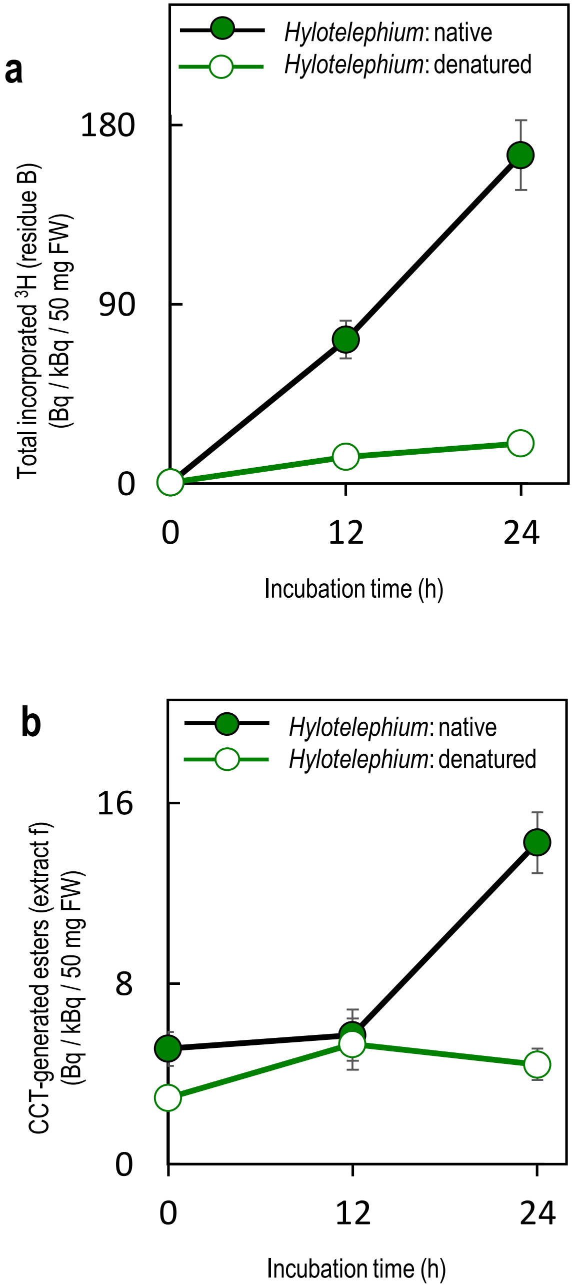

Fig. S2. Time-course of [^{3}H]HHA insolubilisation and ester bond formation in *Hylotelephium* leaf epidermis i*n situ*.

(a) [^{3}H]HHA incorporation into the methanol-insoluble fraction of *Hylotelephium* leaf epidermis *in situ* at pH 5.5. Each native or heat-denatured, 50-mg epidermis sample was incubated with 0.44 kBq [^{3}H]HHA in 300 µl buffer at 20°C. Total methanol-insoluble ^{3}H-labelled products (residue B of Fig. 2) were assayed.

(b) [^{3}H]HHA incorporation into the alkali-releasable material (extract f of Fig. 2; CCT products) of same samples.

Bars indicate standard errors (n = 3).

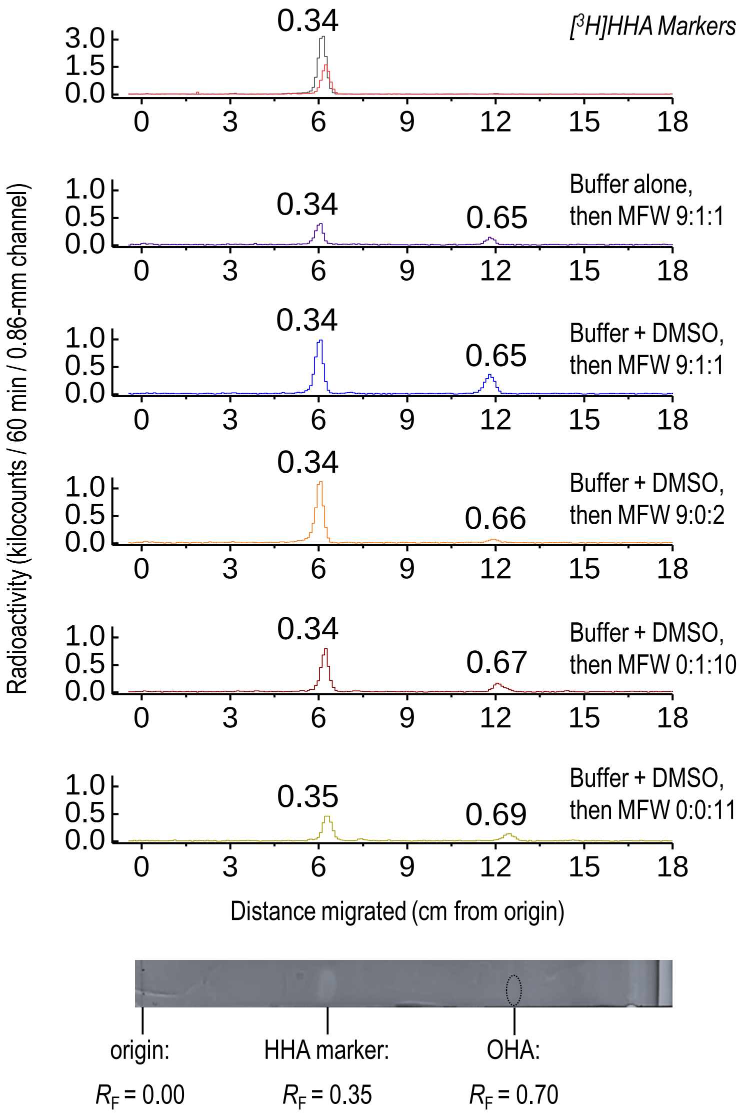

Fig. S3. HHA gives OHA as a by-product during methanolic extraction.

This experiment documents the non-enzymic formation of radiolabelled oxohexadecanoic acid (OHA) as a by-product when authentic [^{3}H]HHA is incubated in the various solvents indicated. Routinely, in our *in-situ* experiments, [^{3}H]HHA was dissolved in a small volume of DMSO then diluted into succinate buffer, and after incubation with epidermis the unincorporated radioactivity was extracted in MFW (9:1:1). In the present experiment we varied these solvents to test which of them contributed to by-product formation. Products were analysed by TLC.

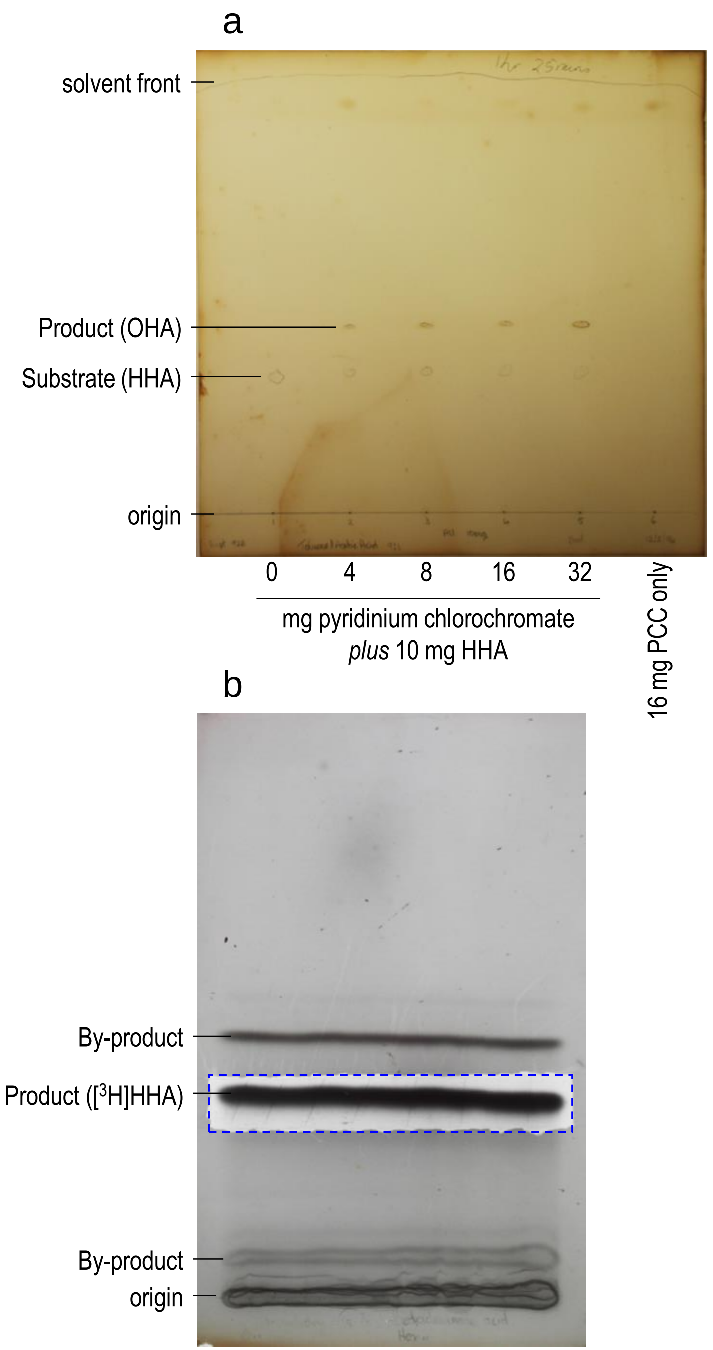

Fig. S4. Preparation of 16-hydroxy-[16-^{3}H]hexadecanoic acid (HHA).

Thin-layer chromatograms (TLCs) illustrating the preparation of [^{3}H]HHA.

(a) Optimising the dose of pyridinium chlorochromate (PCC) required to convert 10 mg of non-radioactive 16-hydroxyhexadecanoic acid (HHA) to 16-oxohexadecanoic acid (OHA).

(b) Autoradiogram of a preparative TLC of the tritiated products obtained after treatment of OHA with $NaB^{3}H_4$; overlaid on the TLC plate. The main radioactive band ([^{3}H]HHA) was cut out for elution.

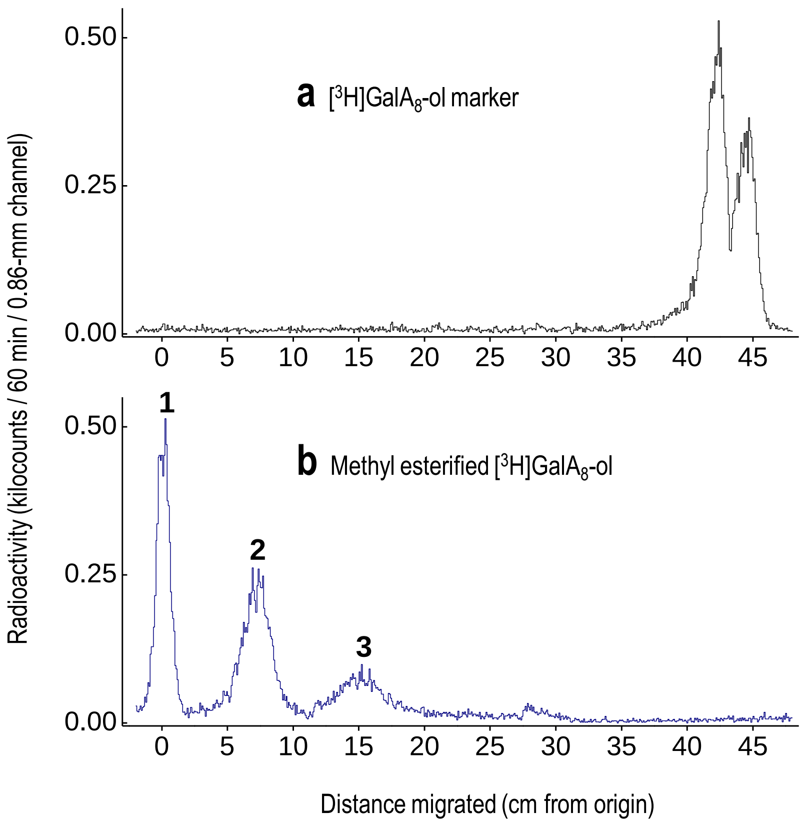

Fig. S5. Preparation of methylesterified [^{3}H]GalA$_8$-ol.

α-(1→4)-[1-^{3}H]GalA$_8$-ol (= reductively tritiated octasaccharide of homogalacturonan) was methylesterified by reaction with methanol in the presence of *N*-ethyl-*N'*-(3-dimethylaminopropyl)carbodiimide (EDC) and *N*-hydroxysuccinimide (NHS) [Sehgal, D. and Vijay, I.K. (1994). A method for the high efficiency of water-soluble carbodiimide-mediated amidation. *Anal. Biochem.* **218**, 87–91]. Substrate and products were resolved by paper electrophoresis, yielding: 1, Me$_8$-[^{3}H]GalA$_8$-ol; 2, Me$_7$-[^{3}H]GalA$_8$-ol; 3, Me$_6$-[^{3}H]GalA$_8$-ol (where Me represents a methylester group). (a) [^{3}H]GalA$_8$-ol substrate; (b) methylesterified products.

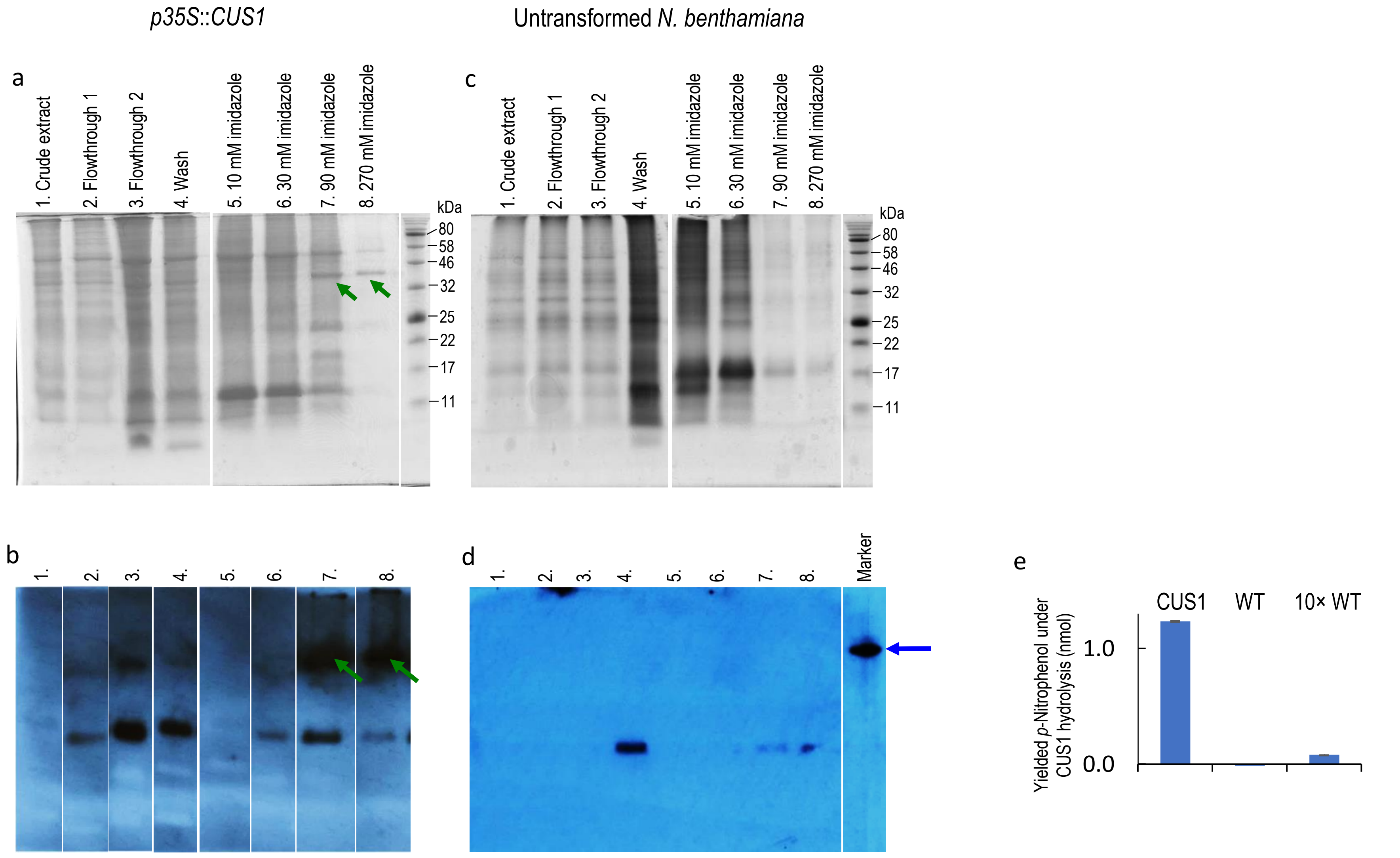

Fig. S6. Purification of *N. benthamiana*-expressed His$_6$-tagged CUS1.

(a, b) SDS-PAGE of fractions from *Nicotiana benthamiana* leaf infiltrated with *Agrobacterium* harbouring *p35S*::*CUS1*. (a) Gel stained with Coomassie Brilliant Blue R-250; (b) bands on a duplicate gel visualised with anti-poly-His–HRP antibody. Green arrows point to the His$_6$-tagged CUS1 (~41 kDa).

(c, d) As a and b but with the corresponding fractions from non-infiltrated *N. benthamiana*. Blue arrow points to a His$_6$-tagged positive control protein [arabidopsis MSI1 (~50 kDa; multicopy suppressor of IRA; Schmitges et al., 2011); kindly donated by Prof. J.W. Goodrich, The University of Edinburgh].

(e) *In-vitro* hydrolysis of fatty-acyl ester by CUS1. *p*-Nitrophenyl palmitate (50 µM) was incubated at 25°C for 3 d in 100 µl aqueous 25 mM succinate (Na^+) buffer (pH 6.0) with 1 µg purified His$_6$-tagged CUS1 or a blank preparation from untransformed *N. benthamiana* (1× and 10× concentration relative to the fresh weight of *N. benthamiana* used for preparation of CUS1). The yield of free *p*-nitrophenol, produced by ester hydrolysis, was assayed by A_{405} at pH 7.15 ± 0.02 and calculated as: rection-mixture A_{405} corrected by subtracting the CUS1-only A_{405} and the *p*-nitrophenyl palmitate-only A_{405}, and converted to nmol by the Beer–Lambert Law. Bar indicates standard error (n=3). $P < 0.01$.

```
CUS1 (Solyc11g006250)            MATPTI---ILSFLL-----------------IFGVAICQSEARAFFVFG 50
Candidate GDSL (Solyc12g017460)  MATFSLTKVSLSVLL-VSL----------FFFSPSSAQTKCNVNSVYQLG
Candidate GDSL (Solyc02g077330)  MLMRLL---IILFLGLVALHDHNGVVQGIEVAVLRKLAAKNNVTCLLVFG

CUS1 (Solyc11g006250)            DSLVDSGNNNY---LATTARADSPPYGIDYPTRRATGRFSNGYNIPDIIS 100
Candidate GDSL (Solyc12g017460)  DSLADAGNVIRTPGASIIFRADRSPYGETFFR-KPTGRFSNGRVITDFIS
Candidate GDSL (Solyc02g077330)  DSSVDPGNNNH---LSTINKANYLPYGMDLNS-QPTGRFSNGKLATDFIV

CUS1 (Solyc11g006250)            QQIGSSESPLPYLDPALTG-QRLLVGANFASAGIGILNDTGIQFINIIRM 150
Candidate GDSL (Solyc12g017460)  QSFK-----LPFLNAYLDRTASFSQGVNFAVAGATALD-TSFWTARNIRL
Candidate GDSL (Solyc02g077330)  EALGYVNMTRAFLDPQINK-VDMLHGISFASAGSGYD-DLTANESNAMTL

CUS1 (Solyc11g006250)            PQQ----LAYFRQYQSRVSGLIGEANTQRLVNQALVLMTLGGNDFVNNYY 200
Candidate GDSL (Solyc12g017460)  PTWNTPLSNQLTWFKSHLKSTCGSKCAENLKNSIVIMGEWGGNDYYNCFF
Candidate GDSL (Solyc02g077330)  AKQ----REYLRHYEIHLSKMVGVDKARETMKNALYILSMGTNDELQNYF

CUS1 (Solyc11g006250)            LVPNSARSRQFSIQDYVPYLIREYRKILMNVYNLGARRVIVTGTGPLGCV 250
Candidate GDSL (Solyc12g017460)  QRK-----QISEVRSYVPFVVAGIMRGIKEVIQLGATRVLVPSIYPLGCL
Candidate GDSL (Solyc02g077330)  LEV--IRSMQYTVEQYQNFLIRSLFTHVKIIHSRGARRLAVVGVPPLGCE

CUS1 (Solyc11g006250)            PAELAQRSRNG-------ECSPELQRAAGLENPQLTQMLQGLNSELGSDV 300
Candidate GDSL (Solyc12g017460)  PLYLTSFPDNNTSGYDQLGCLKSFNDFASYHNRFVVRAMANLQRQFPNVS
Candidate GDSL (Solyc02g077330)  PLIRTIRDDET-------KCDDDLNKVAFTENLKIKRELQALKRLFGIKV

CUS1 (Solyc11g006250)            FIAANTQQMHTNFITNPQAYGFITS--KVACCGQGPYN---GLGLCTPL- 350
Candidate GDSL (Solyc12g017460)  IVYGDFYGSILSLIRNPSSFGENQNTLLSACCGTGGTHNFNFRTVCGGAG
Candidate GDSL (Solyc02g077330)  AYI-DIYSIILEAVQNPQKFSETET--SKGCCGTGTVE---YGESCKGL-

CUS1 (Solyc11g006250)            SNLCPNRDVYAFWDPFHPSERANKIIVQQIMSGTT-ELMNPMNLSTILAM 450
Candidate GDSL (Solyc12g017460)  INACSNPAKYVHWDGIHLTDEAHRRISEVLVRDMLA----KFNCVV----
Candidate GDSL (Solyc02g077330)  -KTCGDRTKFVFWDAVHPSEKMHKIMANEALKAINVDLLD----------

CUS1 (Solyc11g006250)            DSHA 454
Candidate GDSL (Solyc12g017460)  ----
Candidate GDSL (Solyc02g077330)  ----
```

Fig. S7. Protein sequence alignment of CUS1 with two co-expressed tomato GDSL proteins.

The protein sequences encoded by *Solyc12g017460* and *Solyc02g077330* were blasted against CUS1 sequence (*Solyc11g006250*) through Clustal Omega (https://www.ebi.ac.uk/Tools/msa/clustalo/). The ‘GDSL’ (or similar) sequence is residues 50–53. Amino acid residues are highlighted as follows:

Blue: neutral amino acids (M, A, I, L, F, V, W); yellow: cyclic amino acid (P); green: amino acids with polar uncharged side groups (S, T, Q, N); orange: the simplest amino acid (G); pink: sulphur-containing amino acid (c); purple: negative-charged amino acids (E, D); red: positive-charged amino acids (R, K); turquoise: amino acids with polar aromatic side groups (Y, H).
